# Transcriptional and Functional Analysis of CD1c^+^ Human Dendritic Cells Identifies a CD163^+^ Subset Priming CD8^+^CD103^+^ T Cells

**DOI:** 10.1016/j.immuni.2020.06.002

**Published:** 2020-08-18

**Authors:** Pierre Bourdely, Giorgio Anselmi, Kristine Vaivode, Rodrigo Nalio Ramos, Yoann Missolo-Koussou, Sofia Hidalgo, Jimena Tosselo, Nicolas Nuñez, Wilfrid Richer, Anne Vincent-Salomon, Alka Saxena, Kristie Wood, Alvaro Lladser, Eliane Piaggio, Julie Helft, Pierre Guermonprez

**Affiliations:** 1 Centre for Inflammation Biology and Cancer Immunology, The Peter Gorer Department of Immunobiology, School of Immunology & Microbial Sciences, King’s College London, London, UK; 2 Cancer Research UK King’s Health Partner Cancer Centre, King’s College London, London, UK; 3 PSL Research University, Institut Curie Research Center, Translational Immunotherapy Team, INSERM U932, Paris, France; 4 Laboratory of Immuno-oncology, Fundación Ciencia & Vida, Santiago, Chile; 5 PSL Research University, Institut Curie, Department of Biopathology, Paris, France; 6 National Institute of Health Research Biomedical Research Centre at Guy’s and St Thomas’ Hospital and King’s College London, London, UK; 7 Facultad de Medicina y Ciencia, Universidad San Sebastián, Santiago, Chile; 8 Université de Paris, Centre for Inflammation Research, CNRS ERL8252, INSERM1149 Paris, France

**Keywords:** conventional DCs, DC3s, cDC2s, mononuclear phagocytes, inflammatory DCs, monocytes, DC progenitors, T_RM_

## Abstract

Dendritic cells (DCs) are antigen-presenting cells controlling T cell activation. In humans, the diversity, ontogeny, and functional capabilities of DC subsets are not fully understood. Here, we identified circulating CD88^−^CD1c^+^CD163^+^ DCs (called DC3s) as immediate precursors of inflammatory CD88^−^CD14^+^CD1c^+^CD163^+^FcεRI^+^ DCs. DC3s develop via a specific pathway activated by GM-CSF, independent of cDC-restricted (CDP) and monocyte-restricted (cMoP) progenitors. Like classical DCs but unlike monocytes, DC3s drove activation of naive T cells. *In vitro*, DC3s displayed a distinctive ability to prime CD8^+^ T cells expressing a tissue homing signature and the epithelial homing alpha-E integrin (CD103) through transforming growth factor β (TGF-β) signaling. *In vivo*, DC3s infiltrated luminal breast cancer primary tumors, and DC3 infiltration correlated positively with CD8^+^CD103^+^CD69^+^ tissue-resident memory T cells. Together, these findings define DC3s as a lineage of inflammatory DCs endowed with a strong potential to regulate tumor immunity.

## Highlights

• DC3s are phenotypic and functional intermediates between cDC2s and monocytes
• GM-CSF alone, but not FLT3L, supports efficient differentiation of DC3s
• DC3s do not differentiate via cDC (CDP)- or monocyte-restricted (cMoP) progenitors
• DC3s prime T_RM_ cells *in vitro* and correlate with T_RM_ expansion in primary breast cancer

## Graphical Abstract

**Figure:**
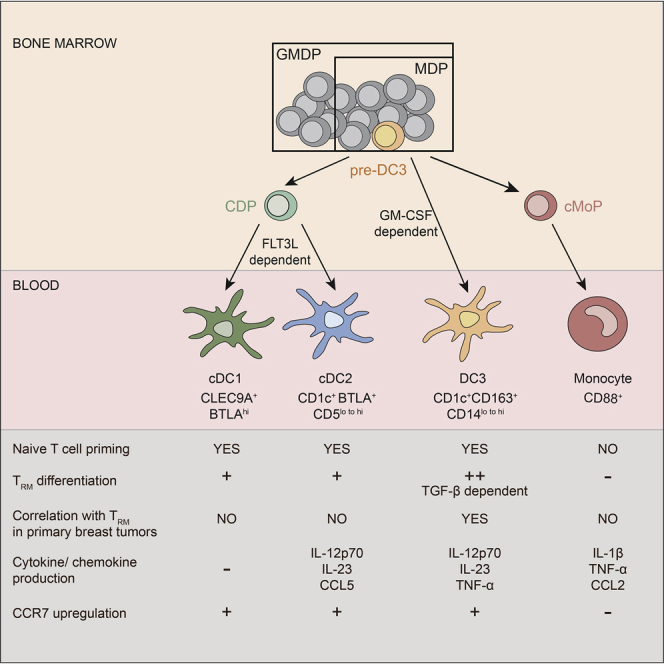

## Teaser

Bourdely et al. identify human CD88^−^CD1c^+^CD163^+^ DC3s as a pro-inflammatory phagocyte lineage sharing features with monocytes and conventional DCs. DC3s efficiently induce differentiation of CD103^+^CD8^+^ T cells *in vitro*, and their infiltration correlates with CD8^+^CD69^+^CD103^+^ T_RM_ accumulation in breast tumors.

## Introduction

Human dendritic cells (DCs) are sentinel cells of the immune system specialized in controlling T cell function (Banchereau and Steinman, 1998; Palucka and Banchereau, 2013; Steinman et al., 2003). The mouse model has brought important concepts to our understanding of DCs and suggests that multiple DC subsets arising from specialized ontogenetic pathways are endowed with specific immune functions (Briseño et al., 2016; Guermonprez et al., 2019; Merad et al., 2013; Murphy et al., 2016).

Definition of human DC subsets is a prerequisite to understanding the division of labor underpinning induction of various types of immune responses. At homeostasis, conventional DCs (cDCs) include cDC1s (CD141^+^XCR1^+^CLEC9A^+^IRF8^+^) and cDC2s (CD1c^+^CD11c^+^CD172a^+^IRF4^+^) (Bachem et al., 2010; Crozat et al., 2010; Heidkamp et al., 2016; Jongbloed et al., 2010; Schlitzer et al., 2013). cDC1s and cDC2s arise through a specialized ontogenetic pathway from a common DC precursor (CDP) (Lee et al., 2015b) or from early IRF8^+^ multipotent lympho-myeloid progenitors (MLPs) (Helft et al., 2017; Lee et al., 2017). Bone marrow progenitors for cDCs generate a common circulating precursor that progressively diverges into pre-cDC1 and pre-cDC2 (Breton et al., 2015, 2016; See et al., 2017). This is further complicated by inclusion of AXL^+^SIGLEC6^+^CD11c^+^CD1c^+^ cells (AS-DCs also called type 5 DCs), which have been proposed to act as precursors for cDCs (pre-cDCs) or a lineage on its own (See et al., 2017; Villani et al., 2017).

An additional layer of complexity in the DC network lies in its responsiveness to perturbations. For instance, inflammation affects hematopoiesis and phagocyte trafficking, resulting in leukocyte mobilization and tissue infiltration. Specifically, inflammation affects DC diversity and triggers mobilization of CD14^+^CD1c^+^ DCs, called inflammatory DCs (iDCs) (Binnewies et al., 2019; Granot et al., 2017; Segura et al., 2012, 2013; Wollenberg et al., 1996; Zaba et al., 2009). The expression of CD1c lectin is shared between iDCs and cDCs. However, CD1c^+^ iDCs also express multiple monocytic markers, such as CD14, CCR2, and FcγRI/CD64. iDCs have been reported in inflamed skin, synovial fluid, ovarian cancer ascites, solid tumor infiltrates, and lymph nodes (Bakdash et al., 2016; Binnewies et al., 2019; Granot et al., 2017; Lavin et al., 2017; Segura et al., 2012, 2013; Wollenberg et al., 1996; Zaba et al., 2009). The developmental pathway of human CD1c^+^CD14^+^ iDCs is poorly understood. *In vitro* studies suggest that iDCs obtained after differentiation of CD14^+^ monocytes in granulocyte macrophage colony-stimulating factor (GM-CSF) and interleukin-4 (IL-4) (Sallusto and Lanzavecchia, 1994) might correspond to *in vivo* iDCs (Granot et al., 2017; Segura et al., 2012, 2013). In this context, IL-4 acts through induction of the transcriptional regulator NCOR2 (Sander et al., 2017). In addition, triggering the aryl hydrocarbon receptor in monocytes supports activation of IRF4-dependent differentiation of iDCs (Goudot et al., 2017). Together, these studies support the prevailing notion that CD14^+^ monocytes act as immediate precursors for iDCs.

Re-evaluation of circulating mononuclear phagocyte diversity has been enabled by single-cell RNA sequencing (scRNA-seq). Recent studies have revealed that a subset of DC-like cells, called DC3s, express mRNA for the CD14 and CD1c genes (Villani et al., 2017). However, this analysis was performed after excluding cells expressing the highest amount of CD14 (Villani et al., 2017). As a consequence, this approach renders a problematic distinction between DC3s and *bona fide* CD14^+^ monocytes (Villani et al., 2017). This discrimination is further complicated by previous reports of CD14^+^CD1c^+^ “inflammatory” DCs recruited at inflammatory sites (Binnewies et al., 2019; Granot et al., 2017; Segura et al., 2012, 2013; Wollenberg et al., 1996; Zaba et al., 2009).

Here we intended to re-evaluate the definition of DC3s using unbiased scRNA-seq and high-dimensional flow cytometry by exploring the full spectrum of CD14 and CD1c expression. In addition, we identify DC3 growth factor requirements and developmental pathways. Finally, we show that DC3s activate CD103^+^ T cells and that DC3 infiltration in human breast tumors correlates with the abundance of CD8^+^CD103^+^CD69^+^ tissue-resident memory (T_RM_) T cells.

## Results

### DC3s Represent a Discrete Subset of CD88^−^CD1c^+^CD163^+^ Cells in Human Peripheral Blood

**Figure 1:**
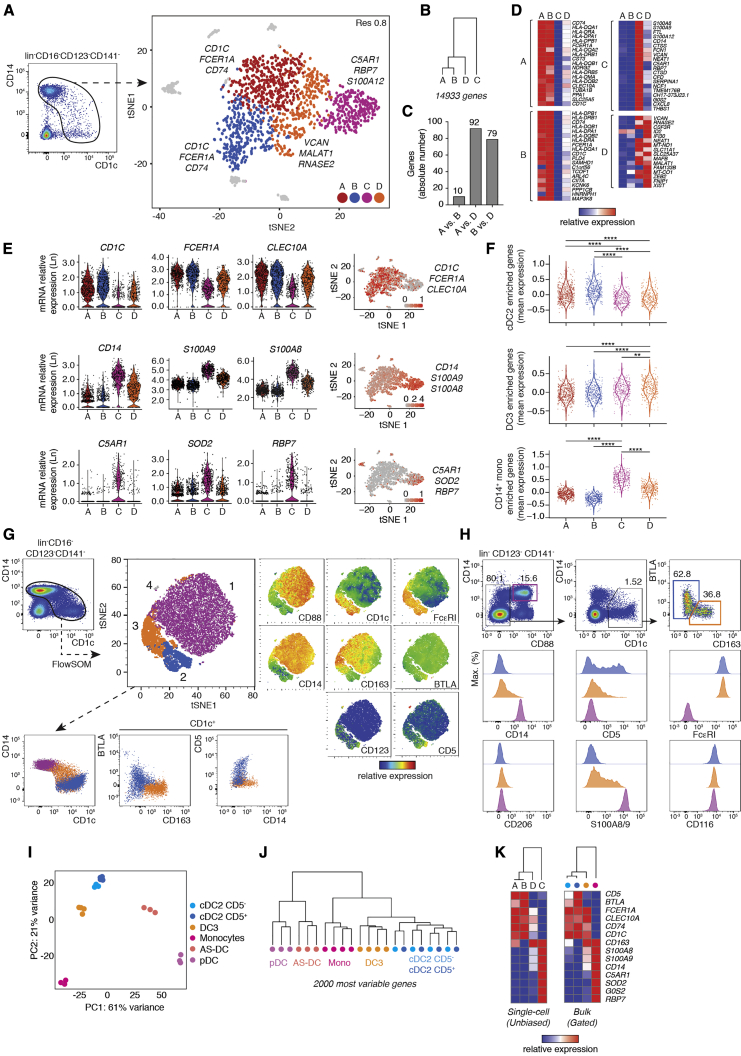
DC3s Are a Discrete Subset of CD88^−^CD1c^+^CD163^+^ Cells in Human Peripheral Blood (A) Gating strategy used to define mononuclear phagocytes expressing CD14 and/or CD1c. Cells expressing CD14 and/or CD1c were sorted by flow cytometry from 3 healthy donors and pooled before scRNA-seq analysis. To improve the resolution of CD1c^+^ subsets, the cellular input was enriched in CD1^high^ cells (Figure S1A). Single cells were isolated using a droplet-based approach and sequenced. Dimensionality reduction of scRNA-seq data was performed using dimensionality reduction (t-distributed stochastic neighbor embedding [tSNE]). Clusters A, B, C, and D were identified using the shared nearest neighbor (SNN) clustering algorithm. Each dot represents an individual cell (n = 1,622). (B) Hierarchal clustering of groups A, B, C, and D based on average gene expression (14,933 genes). (C) Absolute number of differentially expressed genes (DEGs) for pairwise comparisons between groups A, B, and D. (D) Heatmaps displaying relative expression of up to 20 DEGs defining each cluster. (E) Violin plots illustrating expression probability distributions across clusters of representative DEGs (226 total DEGs). Feature plots display the average expression of groups of genes (identified in violin plots) in each cell of the tSNE plot defined in (A). (F) Expression distribution across clusters A, B, C, and D of gene signatures identified by Villani et al. (2017) and Yin et al. (2017). (^∗∗^p < 0.01, ^∗∗∗∗^p < 0.0001, one-way ANOVA test) (G) Identification of 4 subsets within CD14^lo to hi^ CD1c^lo to hi^ cells by unsupervised clustering of flow cytometry data using the FlowSOM algorithm. tSNE and unsupervised clustering were performed using the following markers: CD88, CD1c, FcεRI, CD14, CD163, BTLA, CD123, and CD5. tSNE plots (right) display the relative expression of each marker among the subsets. Dot plots (below) show the expression of specific markers in clusters 1, 2, and 3 when combined in 2-dimensional analysis. (H) Improved gating strategy for identification of cDC2s, DC3s, and CD14^+^ monocytes in circulating PBMCs and histograms showing expression of S100A8/9, FcεRI, CD5, CD14, CD116, and CD206. (I) Principal-component analysis (PCA) for bulk-sequenced mononuclear phagocyte populations as defined in (H). (J) Cluster dendrogram of the different cell types using the 2,000 most variable genes. (K) Heatmaps comparing the relative expression of markers discriminating clusters in scRNA-seq analysis (A, B, C, and D, left) and in bulk RNA-seq analysis on sorted subsets based on the gating strategy defined in (H) (right). See also Figure S1 and Table S1.

To probe the diversity of CD16^−^CD141^−^CD123^−^ blood mononuclear phagocytes, we developed a sorting strategy including all phenotypic intermediates between CD14^hi^CD1c^lo^ and CD14^lo^CD1c^hi^ cells. The proportions between cell populations were compensated to enrich in less abundant CD14^lo^CD1c^hi^ cells (Figure S1A). Flow cytometry-sorted cells isolated from blood were analyzed using a droplet-based scRNA-seq approach (Figure 1A; Figure S1A). We found that cells expressing CD14 and/or CD1c could be separated into four CD33^+^ clusters (A, B, C, and D) (Figure 1A; Figure S1B). Contaminating clusters containing B and T lymphocytes and neutrophils were excluded from the analysis (Figure S1B). Hierarchical clustering performed on averaged single cell expression data within clusters showed that A and B were closer to each other than any of the other subsets (Figures 1B–1D). Cluster D fell between the group of clusters A and B and cluster C (Figure 1B). Classical cDC2 markers, such as C*LEC10A*, *FCER1A*, and major histocompatibility complex (MHC) class II genes (*HLA-DQA*, *HLA-DPA*, *HLA-DRA*, and the MHC class II-associated invariant chain *CD74*) were expressed prominently in clusters A, B, and D (Figures 1D and 1E; Figure S1C; Heidkamp et al., 2016; Lavin et al., 2017; Segura et al., 2013). In contrast, monocytic markers such as *CD14*, *S100A8*, *S100A9*, *S100A12*, and *VCAN* were more expressed in clusters C and D, with higher expression in C compared with D (Figures 1D and 1E). Finally, expression of the C5 receptor *C5AR1* (CD88) was found to be restricted to cluster C together with *SOD2* and *RBP7* (Figures 1D and 1E).

To determine whether our clustering reflects previously published data, we decided to evaluate the expression of gene signatures obtained from Villani et al. (2017). We found that signature genes discriminating cDC2 within CD14^lo^ cells were mostly represented in clusters A and B, confirming their identity (Figure 1F). Likewise, signature genes defining DC3s within CD14^lo^ cells (Villani et al., 2017) and CD14^+^ monocytes were significantly enriched in cluster D and cluster C, respectively (Figure 1F). Of note, genes enriched in DC3s compared with cDC2s (*S100A8*, *S100A9*, and *CD14*) were highly expressed in clusters C and D (Figure 1E; Figure S1E).This underlines the need to integrate a monocyte reference in any comparison aiming to define DC3s. We conclude that scRNA-seq analysis identifies DC3s (cluster D) as a specific subset sharing transcriptional features of cDC2s (clusters A and B) and monocytes (cluster C).

Next we sought to define a flow cytometry-based strategy enabling analysis and prospective isolation of DC3s in blood. To this end, we performed an unsupervised flow cytometry data analysis based on genes identified by scRNA-seq (e.g., CD88, CD14, FcεRI, and CD1c) as well as markers previously associated with cDC2s (e.g., BTLA and CD5) (Yin et al., 2017) and DC3s (e.g., CD163) (Villani et al., 2017), even though they were not detected in the scRNA-seq analysis. We identified three main clusters (1, 2, and 3) together with a rarer cluster (4) of CD5^hi^ cells (Figure 1G; Figure S1D). Cluster 4 appeared to be CD123^hi^ contamination of AS-DCs (Figure S1D). Cluster 1 highly expressed CD88, aligning with monocyte cluster C identified by scRNA-seq. Cluster 2 did not express the monocyte-associated markers CD14 and CD88 but was characterized by expression of CD1c, FcεRI, and B and T lymphocyte attenuator (BTLA) (Figure 1G). In addition, cluster 2 showed heterogeneous expression of CD5 (Figure 1G). Similar to cluster 2, cluster 3 did not express the monocytic marker CD88 and displayed higher amounts of CD1c and FcεRI. However, cluster 3 could be distinguished from cluster 2 by higher expression of CD163 and heterogeneous expression of CD14 (Figure 1G). The unsupervised flow cytometry analysis allowed us to define a simple gating strategy enabling prospective isolation of monocytes (CD88^+^CD14^+^), cDC2s (CD5^+^ and CD5^−^CD88^−^CD1c^+^BTLA^+^CD163^−^), and DC3s (CD88^−^CD1c^+^BTLA^−^CD163^+^) (Figure 1H).

To further validate the flow cytometry-based identification of cell subsets, we performed bulk RNA-seq analysis of sorted monocytes, cDC2s (CD5^+^ and CD5^−^), DC3s, plasmacytoid DCs (pDCs), and AS-DCs (Figure S1E). Principal-component analysis (PCA) indicated that, overall, CD5^+^ and CD5^−^ cDC2s, DC3s, and monocytes separated from pDCs and AS-DCs along the PC1 axis, accounting for 61% variance (Figure 1I). CD5^+^ and CD5^−^ cDC2 clustered closely together, and DC3s separated clearly from cDC2s and monocytes (Figure 1I; Figure S1F). Hierarchical clustering (HC) and differentially expressed gene analysis led to the same conclusion, with DC3s sitting between monocytes and cDC2s (CD5^+^ and CD5^−^) (Figure 1J; Figure S1G). Overall, DC3s are closer to cDC2s than monocytes (Figures 1I and 1J).

Together, this validates that cellular clusters isolated by a flow cytometry-based approach align to clusters identified by unbiased scRNA-seq (Figure 1K).

We conclude that DC3s are a separable entity within CD1c^+^ cells, defined by a distinct gene expression profile, and that they can be prospectively isolated using CD88, CD1c, CD163, and BTLA (Figure S1H). scRNA-seq and bulk gene expression profiling identify markers shared between DC3s and cDC2s (e.g., *CLEC10A* and *FCER1A*) and markers shared between DC3s and monocytes (such as *S100A8*, *S100A9*, *CD14*, and *CD163*).

### DC3s Give Rise to CD14^+^CD1c^+^ DCs Infiltrating Tumors

**Figure 2:**
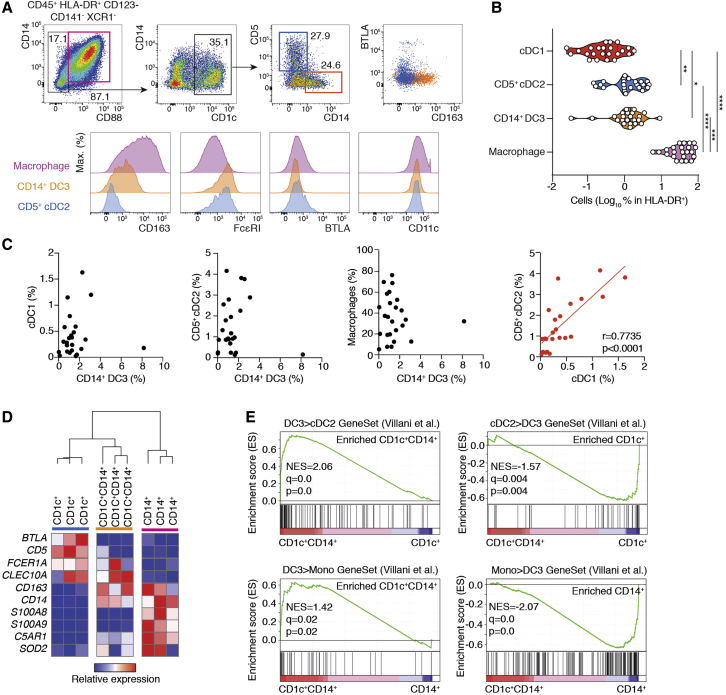
DC3s Infiltrate Human Breast Tumors (A) Representative gating strategy used to define macrophages, CD5^+^ cDC2s, and CD14^+^ DC3s and histograms showing the expression of CD163, FcεRI, BTLA, and CD11c in human breast cancer primary tumors. (B) Violin plot quantifying cDC1, CD5^+^cDC2, CD14^+^ DC3, and CD14^+^CD88^+^ macrophage subsets identified in (A) in human breast cancer primary tumors (n = 25; ^∗^p < 0.05, ^∗∗^p < 0.01, ^∗∗∗^p < 0.001, ^∗∗∗∗^p < 0.0001, one-way ANOVA test). (C) Pearson correlations of the frequencies of macrophages, cDC1s, CD5^+^cDC2s, and CD14^+^ DC3s within HLA-DR^+^ cells in human breast cancer primary tumors (red, significantly correlated p < 0.05; black, not correlated). (D) HC showing the relative expression of markers used for subset identification in Figure 1 in CD1c^+^, CD1c^+^CD14^+^, and CD14^+^ cells from invaded lymph nodes draining human breast cancer primary tumors. (E) GSEA of pairwise comparisons of CD1c^+^CD14^+^ cells with CD1c^+^ or CD14^+^ from invaded lymph nodes draining human breast cancer primary tumors. Gene signatures of blood DC3s compared with cDC2s (DC3 > cDC2) or CD14^+^ monocytes (DC3 > Mono) and, vice versa, of blood cDC2s (cDC2 > DC3) or CD14^+^ monocytes (Mono > DC3) compared with DC3s were used (Villani et al., 2017). See also Figure S2 and Table S2.

Tumor-infiltrating CD14^+^CD1c^+^ DCs have been reported in multiple instances, including ovarian cancer ascites (Segura et al., 2013), breast cancer (Michea et al., 2018), and melanoma (Bakdash et al., 2016; Binnewies et al., 2019). Therefore, we asked whether CD14^+^CD1c^+^ cells would align with DC3s. To this end, we analyzed mononuclear phagocytes infiltrating luminal breast cancer primary tumors. Using the gating strategy described in Figure 1, we found that, after exclusion of CD88^+^ monocytes and macrophages, the remaining CD45^+^HLA-DR^+^CD123^−^CD88^−^ fraction contained cDC1s, CD14^−^CD1c^+^CD163^−^CD5^+^ cDC2s (CD5^+^ cDC2s), and CD14^+^CD1c^+^CD163^+^CD5^−^ DC3s (CD14^+^ DC3s) (Figure 2A; Figure S2A). At this stage, we observed that CD163 and BTLA were particularly susceptible to enzymatic digestion of solid tissue, preventing consistent and reliable quantification of cells throughout the cohort of samples. Hence, we adopted a more restrictive definition of cDC2s and DC3s as CD1c^+^CD14^−^CD5^+^ and CD1c^+^CD14^+^CD5^−^ cells, respectively (Figure 2A). This strategy enabled isolation of CD5^+^ cDC2s and CD14^+^ DC3s in peripheral tissues even though it might result in underestimation of their absolute numbers. Nevertheless, both subsets aligned phenotypically with their blood counterparts (Figures S2B and S2C) and expressed markers reported previously for CD1c^+^CD14^+^ iDCs, such as CD11c and FcεRI (Figure 2A; Segura et al., 2013).

DC3s were consistently identified in 25 samples of primary tumors of clinical stages I, II, and III (Figure S2D). Macrophages represented by far the most abundant population. DC3s outnumbered cDC1s but were on par with *bona fide* CD5^+^ cDC2s (Figure 2B). The relative abundance of DC3s did not correlate with disease progression (Figure S2D) or with macrophages or cDCs (Figure 2C). In contrast, cDC1 infiltration correlated with cDC2s (Figure 2C).

Bulk RNA-seq analysis of CD1c^+^CD14^+^ cells sorted from tumor-invaded lymph nodes indicated that they displayed a similar expression profile as blood DC3s (high expression of cDC2 markers such as *CLEC10A* and *FCER1A* combined with low expression of monocyte-associated markers such as *C5AR1* and *SOD2*) (Figure 2D). Gene set enrichment analysis (GSEA) revealed that CD14^+^CD1c^+^ were more enriched for the DC3 > cDC2 signature compared with CD1c^+^ cells (normalized enrichment score [NES], 2.06; p = 0.0; Subramanian et al., 2005; Villani et al., 2017). In addition, CD14^+^CD1c^+^ were more enriched for the DC3 > monocyte (Mono) signature compared with CD14^+^ cells (NES, 1.42; p = 0.02; Villani et al., 2017; Figure 2E). Conversely, compared with CD1c^+^CD14^+^, the cDC2 > DC3 and Mono > DC3 gene signatures were enriched in CD1c^+^ (NES, −1.57; p = 0.004) and CD14^+^ (NES, −2.07; p = 0.0) cells, respectively (Figure 2E).

We conclude that CD11c^+^FcεRI ^+^CD14^+^CD1c^+^ iDCs infiltrating breast cancer align with DC3s.

**Figure 3:**
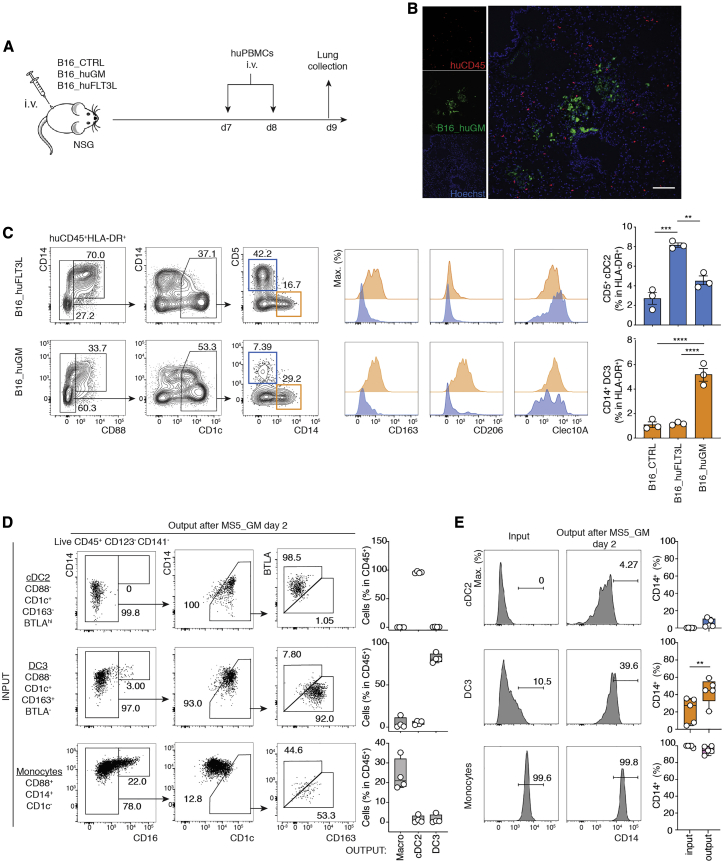
DC3s Give Rise to CD14^+^CD1c^+^ DCs at Inflammatory Sites (A) Experimental model. NSG mice were injected intravenously (i.v.) with B16_CTRL, B16_huFLT3L, or B16_huGM on day 0. On days 7 and 8, 10^8^ human PBMCs were injected i.v. Metastatic lungs were collected on day 9. (B) Pseudocolor images of B16_huGM (green) metastatic lung on day 9 post-injection, stained for human CD45 (red). Nuclei were stained with Hoechst (blue). Scale bar, 100 μm. (C) Gating strategy for cDC2 and DC3 identification in B16_huGM and B16_huFLT3L metastatic mouse lung and histograms showing the expression of CD163, CD206, and Clec10A. The bar graph summarizes the frequency of cDC2s and DC3s among total HLA-DR^+^ cells in metastatic B16_CTRL, B16_huFLT3L, or B16_huGM mouse lungs (n = 3 independent mice; ^∗∗^p < 0.01, ^∗∗∗^p < 0.001, ^∗∗∗∗^p < 0.0001, one-way ANOVA test). (D) Flow cytometry analysis of flow cytometry-sorted cDC2s, DC3s, and CD14^+^ monocytes after 2 days of culture with MS5 stromal cells expressing human GM-CSF (MS5_GM). Bar graphs show the frequency of output cells among total huCD45^+^ cells (n = 4–5 healthy donors). (E) Histograms showing CD14 expression on cDC2s, DC3s, and CD14^+^ monocytes before and after 2 days of coculture with MS5_GM and bar graphs summarizing the frequency of CD14 expression within each cell type (n = 5 healthy donors, ^∗∗^p < 0.01, Mann-Whitney two-tailed t test). See also Figures S3 and S4 and Table S1.

Inflammatory cues promoting mobilization of CD14^+^CD1c^+^ cells at the site of inflammation are not fully defined. Mouse studies define GM-CSF as a likely candidate (Mach et al., 2000; Menezes et al., 2016). For this reason, we decided to test whether GM-CSF was sufficient to mobilize human CD14^+^CD1c^+^ DCs in a humanized mouse metastatic lung model. We generated B16 mouse melanoma engineered to overexpress human GM-CSF (B16_huGM) or FLT3L (B16_huFLT3L) (Figure S3A). Immunodeficient NOD.Cg-*Prkdc*^*scid*^ *Il2rg*^*tm1Wjl*^/SzJ (NSG) mice were injected intravenously with B16 control (CTRL), B16_huGM, or B16_huFLT3L (Figure 3A). Lung metastasis-bearing mice were engrafted with human peripheral blood mononuclear cells (PBMCs). Two days later, human CD45^+^ leukocytes were found in lung tumor foci and juxta-tumor areas (Figure 3B; Figures S3B and S3C). Flow cytometry analysis of metastatic lungs showed that FLT3L, but not GM-CSF, promotes expansion of CD1c^+^CD5^+^ cells aligning with blood cDC2s (Figure 3C, blue). In contrast, GM-CSF, but not FLT3L, led to accumulation of CD1c^+^CD14^+^ cells aligning with blood DC3s (Figure 3C, orange; Figure S3D, orange). As shown for circulating peripheral blood subsets in Figure 1, FLT3L-dependent cDC2s and GM-CSF-dependent DC3s shared expression of Clec10A, CD11c, and FcεRI (Figure 3C; Figure S3E). All tumor-bearing lungs contained some monocytes and/or macrophages (CD14^+^CD88^+^) (Figure 3C; Figure S3B). To further establish alignment of GM-CSF-mobilized DC3s, we performed an unbiased scRNA-seq on human CD45^+^HLA-DR^+^ cells expressing CD14 and/or CD1c (Figures S3F and S3G). Two major clusters could be identified. Cluster 0 was characterized by expression of genes associated with DC3s and cDC2s, such as *CLEC10A*, *FCER1A*, and *CD74* (Figures S3H and S3I), and was enriched in DC3 differentially expressed transcripts (Villani et al., 2017; Figure S3J). Conversely, cluster 1 expressed markers defining monocytes (*SOD2*, *C5AR1*, and *G0S2*) and *CXCL2*, *CXCL3*, and *CXCL8* chemokines (Figures S3H and S3I). We conclude that GM-CSF drives mobilization of CD14^+^CD1c^+^ cells aligning phenotypically with circulating DC3s.

We next wondered whether GM-CSF could induce *trans*-differentiation of circulating cDC2s or monocytes into CD14^+^CD1c^+^ DC3s. To test this hypothesis, cDC2s, DC3s, and monocytes were sorted by flow cytometry from blood and cocultured *in vitro* in the presence of GM-CSF-expressing stromal cells (MS5_GM). After 2 days, cDC2s did not acquire CD163 or CD14 expression (Figure 3D). In contrast, DC3s did upregulate CD14 (Figures 3D and 3E). We found that monocytes differentiated into CD16^+^ and CD16^−^ macrophages (Figure 3D). Importantly, CD88^+^CD1c^−^CD14^+^ monocytes did not give rise to CD1c^hi^CD163^hi^ DC3s (Figure 3D). Addition of IL-4 to GM-CSF culture did not affect the outcome (Figure S4A). The lack of *trans*-differentiation of cDC2s or monocytes into DC3s was also confirmed *in vivo* upon adoptive transfer into immunodeficient NSG mice carrying GM-CSF-expressing tumors (Figures S4B and S4C). Overall, we conclude that CD88^−^CD14^+^CD1c^+^ cells can differentiate from DC3s independent of cDC or monocytic lineages.

### GM-CSF Stimulates Differentiation of CD34^+^ Hematopoietic Progenitors into DC3s *In Vitro*

**Figure 4:**
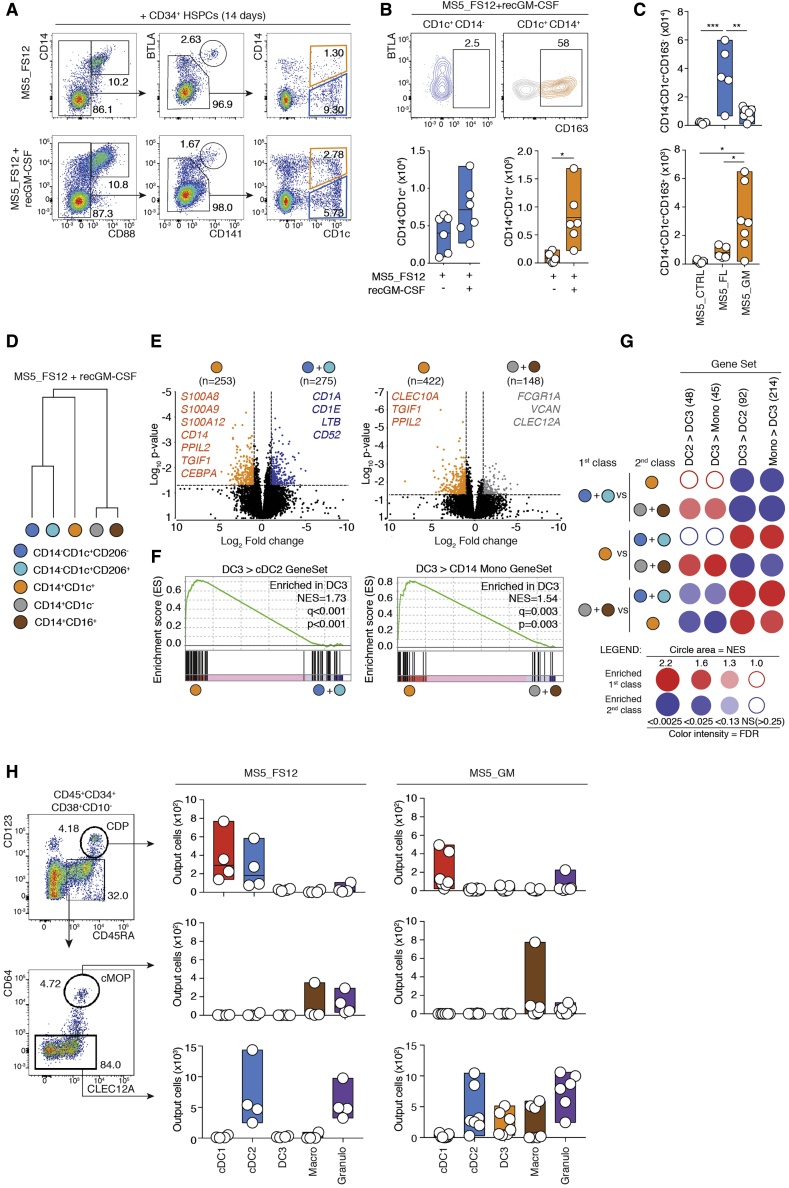
DC3s Differentiate from Hematopoietic Progenitors upon GM-CSF Exposure Independent of Mono-Committed Progenitors (cMoPs) or cDC-Committed Progenitors (CDPs) (A) Flow cytometry analysis of cord blood-derived CD34^+^ HSPCs cultured on stromal cells expressing human FLT3L, SCF, and CXCL12 (MS5_FS12) with or without human recombinant GM-CSF (MS5_FS12+recGM-CSF) for 14 days. (B) Flow cytometry plots of BTLA and CD163 expression within CD1c^+^CD14^−^ and CD1c^+^CD14^+^ cells identified in (A). Bar graphs summarize the absolute numbers of differentiated CD1c^+^CD14^−^ cDC2s and CD1c^+^CD14^+^ DC3s (a line represents the median; n = 6 independent cord blood donors, ^∗^p < 0.05, Wilcoxon test). (C) Bar graphs summarizing the absolute numbers of CD1c^+^CD14^−^CD163^−^ cDC2s and CD1c^+^CD14^+^CD163^+^ DC3s differentiated from cord blood-derived CD34^+^ HSPCs cocultured for 14 days with stromal cells expressing human FLT3L (MS5_FL), GM-CSF (MS5_GM), or neither (MS5_CTRL) (a line represents the median; n = 5–7 independent cord blood donors, ^∗^p < 0.05, ^∗∗^p < 0.01, ^∗∗∗^p < 0.001, Wilcoxon test). (D) HC based on 19,791 protein-coding genes of *in-vitro-*generated subsets differentiated from CD34^+^ HSPCs cultured with MS5_FS12 supplemented with human recombinant GM-CSF (MS5_FS12+recGM-CSF). Each dot represents an average of three donors. (E) Volcano plots showing the DEGs between *in-vitro-*generated DC3s cells (orange) compared with cDC2s (blue and turquoise, left plot) or macrophages (gray and brown, right plot). Genes with Log_2_(fold change, FC) > ±2 and a false discovery rate (FDR)-adjusted p value of less than 0.05 were considered significant. (F) GSEA of pairwise comparisons of DC3s with cDC2s or macrophages generated i*n vitro*. Gene signatures (gene set) defining genes upregulated in blood DC3s compared with cDC2s (DC3 > cDC2) or blood DC3s compared with CD14^+^ monocytes (DC3 > CD14 Mono) were used (Villani et al., 2017) (NES, normalized enrichment score). (G) BubbleMap summarizing the enrichment of defined gene sets in pairwise comparisons of *in-vitro-*differentiated DC3s versus *in vitro* cDC2s or *in vitro* macrophages. Gene signatures (gene sets) of blood DC3s compared with cDC2s (DC3 > cDC2) or CD14^+^ monocytes (DC3 > Mono) and, vice versa, of blood cDC2s (cDC2 > DC3) or CD14^+^ monocytes (Mono > DC3) compared with DC3s were used (Villani et al., 2017). (H) Flow cytometry analysis of cord blood-derived CDPs, cMoPs, and GMDPs cultured for 7 days with MS5_FS12 or MS5_GM. Bar graphs summarize the absolute number of differentiated cells from each progenitor (a line represents the median, n = 4–7 independent cord blood donors). See also Figure S5 and Table S1.

We next wondered how DC3s would differentiate from bone marrow progenitors. To this end, we sought to define a culture system capable of generating DC3s together with cDC2s and macrophages. We cocultured human cord blood-derived CD34^+^ hematopoietic stem and progenitor cells (HSPCs) with stromal cells engineered to overexpress cDC-promoting factors (membrane-bound FLT3L together with stem cell factor (SCF) and CXCL12 [MS5_FS12]; Anselmi et al., 2020) in the presence or absence of GM-CSF (Figure 4A; Figure S5A). We found that GM-CSF increased differentiation of CD1c^+^CD14^+^CD163^+^ cells (orange) phenotypically aligning with blood DC3s (Figures 4A and 4B). CD1c^+^CD14^−^ cells did not express CD163, suggesting that they mostly align with cDC2s. In addition, CD163 expression was restricted to CD1c^+^CD14^+^ cells (Figure 4B). GM-CSF alone (MS5_GM), but not FLT3L, was sufficient to induce differentiation of DC3s *in vitro* (Figure 4C). In contrast, FLT3L (MS5_FL) was sufficient to induce differentiation of cDC2s (Figure 4C). We conclude that cDC2s and DC3s have distinct growth factor requirements.

We next intended to determine whether the transcriptional landscape of *in-vitro*-generated DC3s from stromal cell cocultures aligned with their *in vivo* counterparts. Bulk RNA-seq analysis of *in-vitro*-generated cells showed that CD14^+^CD1c^+^ DC3s (orange) sat between cDC2s (blue and turquoise) and macrophages (gray and brown) (Figure 4D). Of note, CD1c^+^CD14^−^ cells generated *in vitro* were heterogenous for CD206 expression and were therefore analyzed as two independent subsets (CD1c^+^CD206^−^ [blue] and CD1c^+^CD206^+^ [turquoise]; Figure S5B). However, both subsets displayed very similar transcriptomes regardless of CD206 expression, and the CD1c^+^CD14^−^CD206^+^ and CD1c^+^CD14^−^CD206^−^ fractions strongly resembled circulating cDC2s (Anselmi et al., 2020).

Further analysis of differentially expressed genes (DEGs) showed that *in vitro* DC3s differed from *in vitro* cDC2s by expression of monocyte-associated markers such as *S100A8*, *S100A9*, *S100A12*, and *CD14* (Figure 4E; Figure S5C). Conversely, *in vitro* cDC2s displayed higher expression of *CD1C*, *LAMP3*, *CD52*, and *LTB*, as reported for lung cDC2s (Lavin et al., 2017; Figure 4E; Figure S5C). Using the GSEA methodology (Subramanian et al., 2005), we found that the set of genes upregulated in primary DC3s compared with cDC2s (DC3 > cDC2 from Villani et al., 2017) was enriched in GM-CSF-dependent *in vitro* DC3s compared with *in vitro* cDC2s (Figure 4F). Of note, GM-CSF-exposed cDC2s did not convert to DC3s (Figures S5D and S5E). We found that markers common for primary cDC2s and DC3s (*CLEC10A*, Figure 1) were higher in *in vitro* DC3s compared with *in vitro* macrophages (Figure 4E; Heidkamp et al., 2016). Conversely, *in vitro* macrophages expressed more *FCGR1A*, *C5AR1* (CD88), *CXCL8*, *CXCL1*, *CXCL2*, *CXCL3*, *CCL2*, *CCL3*, and *CCL7* compared with *in vitro* DC3s (Ruffell et al., 2009; Figure 4E; Figures S5C and S5F). Genes upregulated in primary DC3s compared with CD14^+^ monocytes (DC3 > CD14^+^ Mono from Villani et al., 2017) were significantly enriched in *in vitro* DC3s compared with macrophages (Figures 4F and 4G). We conclude that GM-CSF drives, *in vitro*, the differentiation of DC3s aligning to primary blood DC3s.

### DC3s Develop via a Differentiation Pathway Independent of CDPs and cMoPs

The developmental relationship between DC3s and cDCs or monocyte lineages is not known. A classical view defines the development of phagocytes as a stepwise and ordered loss of developmental potential concomitant with lineage commitment. Historically, this process has been identified using prospective isolation of progenitor populations of decreasing potential. Specifically, early granulocyte-monocyte and DC progenitors (GMDPs) carries a tri-lineage potential (Lee et al., 2015b). Loss of neutrophil potential defines monocyte and DC progenitors (MDPs) (Lee et al., 2015b). Loss of monocyte potential defines CDPs, which generate cDCs via a pre-cDC intermediate (Breton et al., 2015, 2016; Lee et al., 2015b; Naik et al., 2007; Onai et al., 2007; See et al., 2017). Finally, loss of DC and neutrophil potential defines monocyte-committed progenitors (cMoPs) (Kawamura et al., 2017). Having established the growth factor requirements for DC3 development, we decided to test the contribution of CDPs and cMoPs to generation of DC3s. Flow cytometry-sorted CDPs and cMoPs and the remaining GMDP-containing fraction (CD34^+^CD38^+^CD45RA^+^CD123^−^CD64^−^) isolated from cord blood-derived HSPCs (see the gating strategy in Figure S5G) were cocultured with stromal cells supporting cDC (MS5_FS12) or DC3 (MS5_GM) differentiation.

Flow cytometry analysis after 7 days of culture showed that CDP gave rise exclusively to cDC1s and cDC2s in MS5_FS12 cocultures, as described previously (Figure 4H; Figure S5H; Lee et al., 2015b). cMoP cultures gave rise solely to CD14^+^CD1c^−^ cells, as described previously (Figure 4H; Figure S5H; Kawamura et al., 2017). As expected, the GMDP-containing fraction gave rise to granulocytes, macrophages, and cDC2s. Importantly, the GMDP-containing fraction also gave rise to DC3s in MS5_GM coculture (Figure 4H; Figure S5H). Therefore, we asked whether DC3s would arise directly from a multipotent progenitor or via formation of an intermediate DC3-committed progenitor devoid of any other lineage potential.

**Figure 5:**
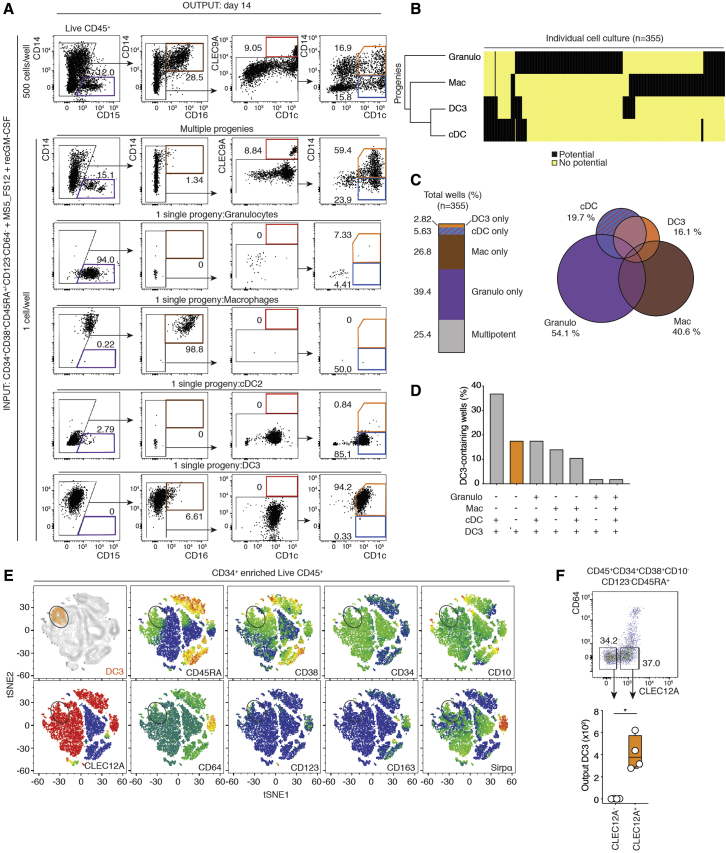
Single-Cell Analysis of DC3 Commitment (A) Flow cytometry analysis of bulk (500 cells) or single CD34^+^CD38^+^CD123^−^CD64^−^ progenitor cells cocultured for 14 days with MS5_FS12 supplemented with recombinant human GM-CSF (MS5_FS12+recGM-CSF). Flow cytometry plots resulting from single CD34^+^CD38^+^CD123^−^CD64^−^ progenitor cells with different potentials are shown as representative examples (n = 355 cells from 2 independent experiments). (B) HC of lineage potential from single CD34^+^CD38^+^CD123^−^CD64^−^ progenitor cells (n = 355). (C) Bar graph and Venn diagram summarizing the frequency of the potential of mono-, bi-, tri-, or multipotent individual CD34^+^CD38^+^CD123^−^CD64^−^ cells within the total wells analyzed (n = 355). (D) Bar graphs summarizing the frequency of mono-, bi-, tri-, or multipotent individual CD34^+^CD38^+^CD123^−^CD64^−^ cells among DC3-generating progenitors only. An orange bar represents the frequency of DC3-restricted progenitors. (E) Cell surface phenotype of DC3-restricted progenitors before differentiation cultures inferred by index flow cytometry sorting. tSNE plots display an overlay of total CD45^+^ cells (gray) and DC3-restricted progenitor cells (orange) (top left). Shown is relative expression of the markers CD45RA, CD38, CD34, CD10, Clec12A, CD64, CD123, CD163, and SIRPα. (F) Validation experiment for identification of Clec12A as a marker for DC3-committed progenitors. Shown is flow cytometry analysis of bulk-sorted CD34^+^CD38^+^CD45RA^+^CD123^−^CD64^−^Clec12A^−^ and Clec12A^+^ cells. 500 cells were cocultured with MS5_GM for 7 days. The bar graph summarizes the number of differentiated DC3s from each bulk population (n = 4 healthy donors, ^∗^p < 0.05, Mann-Whitney two-tailed t test). See also Figure S5.

To address this question in unbiased settings, we developed a single cell culture of CD34^+^CD38^+^CD123^−^CD64^−^ progenitors distinct from CDPs or cMoPs (Figure 5A). We chose to combine MS5_FS12 with soluble GM-CSF for two reasons: (1) GM-CSF alone did not support growth of individual progenitors (Figure S5I), and (2) MS5_FS12 coculture was found to more efficiently support cDC and monocyte differentiation (Figure 4G).

Flow cytometry analysis of 14-day progeny of individual progenitor cultures revealed multiple patterns of developmental potential (Figure 5A). Overall, the granulocyte potential was segregated from the potential for mononuclear phagocytes (Figure 5B). We found that only 0.3% of progenitors could differentiate into all four lineages corresponding to the GMDP functional definition (Figure 5D). Most individual progenitors gave rise to single lineage progeny (Figures 5C and 5D). Unipotent wells containing only neutrophils represented the most abundant outcome (39.4%), followed by macrophage-only (26.8%) and cDC-only (5.63%) wells (Figure 5C). Most individual progenitors endowed with DC3 potential had multi-lineage potential. DC3 potential was more associated with mononuclear phagocytes rather than granulocyte potential (61.4% and 19.3% of DC3-containing wells, respectively) (Figure 5D). Importantly, we also identified a minor fraction of individual progenitors giving rise exclusively to DC3s (Figures 5B–5D).

We next aimed to further define the cell surface phenotype of progenitors endowed with DC3 potential. To this end, we attempted to establish correlations between the cell surface phenotype of single sorted cells (inferred from index flow cytometry sorting) and their developmental potential. *A posteriori* identification of the cell surface phenotype of DC3-committed progenitors revealed that they had a CD34^+^CD38^+^CD45RA^int^CD123^−^CD64^−^SIRPα^lo^CD10^−^Clec12A^+^ phenotype (Figure 5E). As validation, we showed that the potential for DC3s lay in the Clec12A^+^ population of the GMDP-containing fraction (Figure 5F). We conclude that DC3s can develop via a DC3-restricted intermediate distinct from cDC-restricted CDPs or monocyte-restricted cMoPs. Even though the existence of a DC3-commited unipotent progenitor is not formally proven, our data are compatible with the notion that DC3 specification arises downstream of MDP.

### Activated DC3s Induce Priming of Naive T Cells and Differentiation of CD103^+^ T Cells

**Figure 6:**
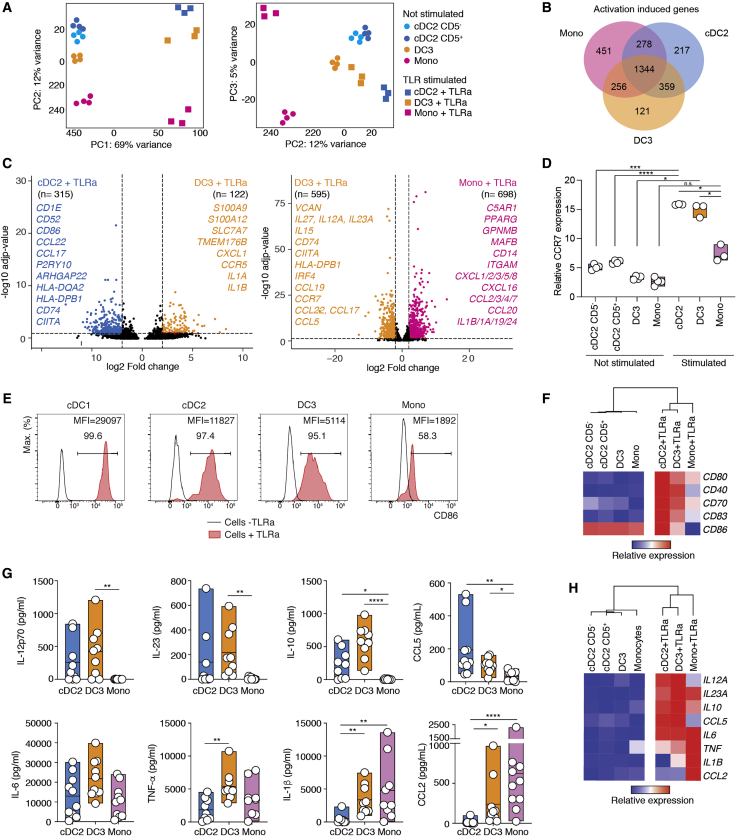
DC3s Respond to TLR Stimulation We performed bulk RNA-seq analysis of BTLA^+^CD5^+^ and BTLA^+^CD5^−^ cDC2s, DC3s, and monocytes sorted as shown in Figure 1H and stimulated overnight (16 h, 3 donors) or not (4 donors) with a TLR agonist cocktail (25 μg/mL poly(I:C), 1 μg/mL R848, and 10 ng/mL LPS). For activation of cDC2s, BTLA^+^CD5^+^ and BTLA^+^CD5^−^ were pooled. (A) PCA analysis for all genes. (B) Venn diagram summarizing the number of activation-induced DEGs upregulated in stimulated compared with unstimulated cells within each cell population. (C) Volcano plots showing DEGs between TLR agonist-stimulated DC3s compared with TLR agonist-stimulated cDC2s or TLR agonist-stimulated monocytes. Genes with Log_2_(FC) > ±2 and a FDR-adjusted p value of less than 0.05 were considered significant. (D) Bar graph summarizing relative CCR7 gene expression within TLR-agonist stimulated or unstimulated mononuclear phagocyte populations (n = 3–4; a line represents the median; ^∗^p < 0.05, ^∗∗^p < 0.01, ^∗∗∗^p < 0.001, ^∗∗∗∗^p < 0.0001, one-way ANOVA test). (E) Histograms showing the frequency and median of fluorescence intensity (MFI) of CD86 on TLR agonist-stimulated or unstimulated mononuclear phagocyte populations. (F) Heatmap showing the relative gene expression of selected costimulatory molecules on TLR agonist-stimulated or unstimulated mononuclear phagocyte populations. (G) Quantification of cytokines and chemokines secreted by cDC2s, DC3s, and CD14^+^ monocytes in response to overnight stimulation with a cocktail of TLR agonists (n = 9 healthy donors; a line represents the median; ^∗^p < 0.05, ^∗∗^p < 0,01, ^∗∗∗∗^p < 0,0001, one-way ANOVA test). (H) Heatmap showing the relative gene expression of cytokines and chemokines analyzed in (G) within TLR-agonist stimulated or unstimulated mononuclear phagocyte populations. See also Figure S6 and Table S1.

We next aimed to understand the immunological function of DC3s and to compare it with cDC2s and monocytes. First, we decided to test the responsiveness of DC3s, cDC2s, and monocytes to a cocktail of Toll-like receptor (TLR) agonists. cDC2s, DC3s, and monocytes were sorted by flow cytometry from blood and stimulated overnight *ex vivo*. PCA analysis of the total transcriptome of unstimulated and stimulated populations evidenced that all subsets underwent a certain degree of convergence in their transcriptome (Figure 6A). In support of this, we found an important overlap in the set of activation-induced genes defined for each subset (1,344 genes; Figure 6B). Despite the relative convergence of activated cells, we found that overnight activation did not compromise cell surface discrimination of DC3s from cDC2s and monocytes (Figure S6A). Indeed, TLR-activated DC3s could still be discriminated from TLR-activated cDC2s by 437 DEGs or from TLR-activated monocytes by 1,293 genes (Figure 6C). The same was true for the pairwise comparison of activated cDC2s and circulating DC3s (Figure S6B). In sum, we conclude that innate activation does not trigger conversion of cDC2s or monocytes into DC3s despite induction of a common transcriptional response to TLR stimulation.

From the perspective of adaptive immunity, activated DC3s shared a lot of common features with activated cDC2s but less with activated monocytes: (1) stimulated DC3s and cDC2s upregulated *CCR7* upon activation (Figure 6D), potentially enabling their ability to migrate toward T cell zones; (2) activated DC3s and cDC2s upregulated cell surface co-stimulatory molecules (*CD80*, *CD86*, *CD70*, and *CD40*; Figure 6E and 6F); (3) activated DC3s and cDC2s efficiently increased the expression of T cell-attracting chemokines such as CCL5 (Figures 6G and 6H), *CCL19*, *CCL17*, *CCL22*, *CXCL9*, *CXCL10*, *CXCL11*, and *CXCL13* (Figure S6C); and (4) activated DC3s and cDC2s produced higher amounts of IL-12p70, IL-23, *IL27*, and IL-10 (Figures 6G and 6H; Figure S6E).

In addition, activated DC3s shared some common features with activated monocytes that are less pronounced in activated cDC2s: (i) activated DC3s and monocytes secreted more inflammatory cytokines, such as tumor necrosis factor alpha (TNF-α) and IL-1β (Figures 6G and 6H); and (2) activated DC3s and activated monocytes upregulated inflammatory chemokines, such as CCL2 (Figures 6G and 6H), *CCL1*, and *CCL3* (Figure S6C) or granulocytes attracting CXCL1, *CXCL3*, and *CXCL5* (Figures S6C and S6D).

Collectively, our data suggest that the transcriptome and secretome of activated DC3s, unlike the ones of activated monocytes, are consistent with a function in priming of naive T cells.

**Figure 7:**
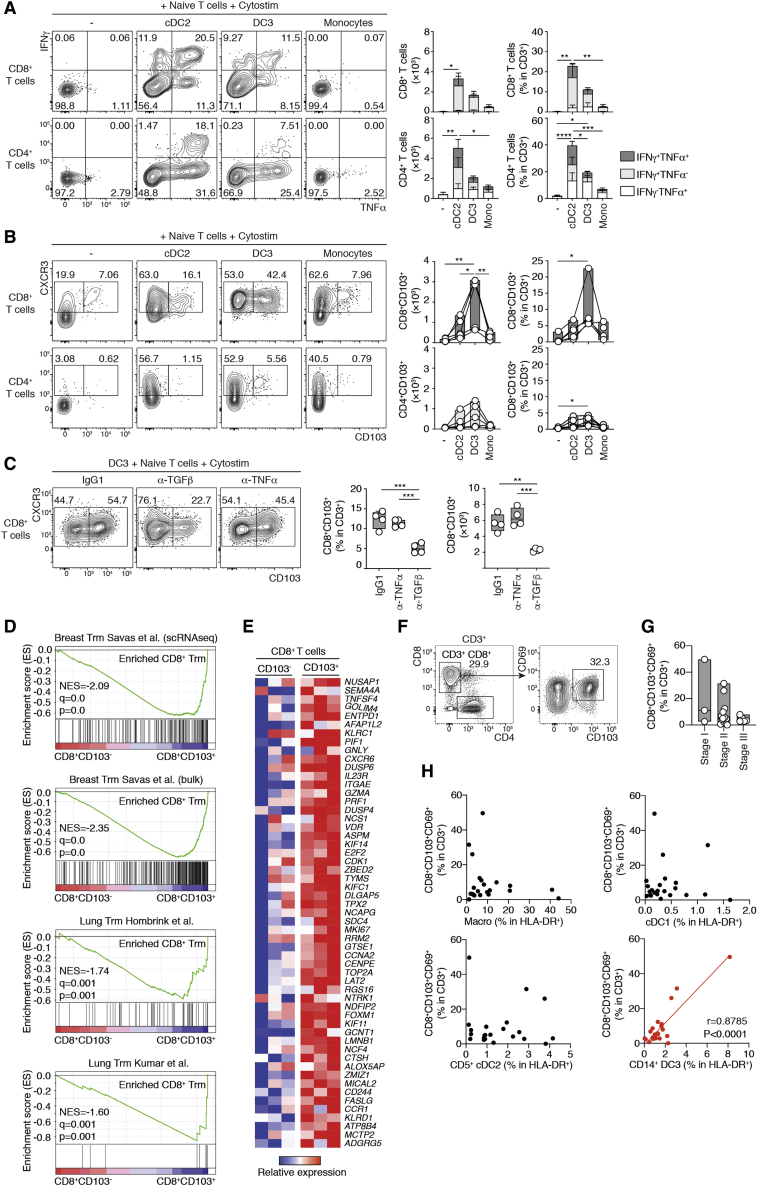
DC3s Prime Naive T Cells and Drive Acquisition of the CD103^+^ T_RM_ Phenotype (A and B) Representative flow cytometry plots and quantification of CD4^+^ and CD8^+^ naive T cells cultured for 5 days with flow cytometry-sorted blood cDC2s, DC3s, or CD14^+^ monocytes after overnight activation with TLR agonists (25 μg/mL poly(I:C), 1 μg/mL R848, and 10 ng/mL LPS) in the presence of a synthetic superantigen (Cytostim). Absolute numbers and frequencies of cytokine-producing and other activated T cells (A) and CD103^+^ T cells (B) are shown (n = 5 healthy donors in 5 independent experiments; a line represents the median; ^∗^p < 0.05, ^∗∗^p < 0.01, ^∗∗∗^p < 0.001, ^∗∗∗∗^p < 0.0001, one-way ANOVA test). (C) Representative flow cytometry plots and quantification showing CD103 expression on CD8^+^ naive T cells cocultured with blood DC3s sorted by fluorescence-activated cell sorting (FACS) in the presence of 10 μg/mL of neutralizing antibodies against TNF-α or TGF-β or an isotype CTRL (n = 4 healthy donors in 3 independent experiments; a line represents the median; ^∗^p < 0.05, ^∗∗^p < 0.01, ^∗∗∗^p < 0.001, ^∗∗∗∗^p < 0.0001, one-way paired ANOVA test). (D and E) Bulk RNA-seq analysis of CD8^+^CD103^−^ T cells (n = 3) and CD8^+^CD103^+^ T cells (n = 3) sorted by flow cytometry after 5 days *in vitro* coculture of naive blood CD8^+^ T cells with blood DC3s activated overnight by TLR agonists. (D) GSEA of pairwise comparisons of CD8^+^CD103^+^ T cells with CD8^+^CD103^−^ T cells. Gene signatures (gene set) defining genes upregulated in breast or lung CD103^+^CD69^+^CD8^+^ T_RM_ cells were used (Hombrink et al., 2016; Kumar et al., 2017; Savas et al., 2018). (E) Heatmap displaying 56 representative genes significantly upregulated in CD8^+^CD103^+^ cells compared with CD8^+^CD103^−^ induced by blood DC3s *in vitro* (of 205 DEGs). Selected genes are shared with at least one of the previously reported gene signatures defining human T_RM_ cells (Hombrink et al., 2016; Kumar et al., 2017; Savas et al., 2018). (F–H) Correlative analysis of T_RM_ cell content in luminal breast cancer primary tumors. (F) Representative flow cytometry plots showing the gating strategy for CD103^+^CD69^+^CD8^+^ T cells in 21 human luminal breast cancer primary tumors. (G) Quantification of CD103^+^CD69^+^CD8^+^ T cells in different stages of human breast tumors (stage I, n = 3; stage II, n = 13; stage III, n = 5). (H) Pearson correlation of the frequencies of the macrophages and cDC1, CD1c^+^ CD14^−^, and CD1c^+^ CD14^−^ cells and the frequencies of CD103^+^CD69^+^CD8^+^ T cells in human breast cancer primary tumors (red, significantly correlated p < 0.05; black, not correlated). See also Figure S7 and Tables S1 and S2.

To directly test the T cell priming capabilities of DC3s, we performed 5-day cocultures of flow cytometry-sorted, activated DC3s, cDC2s, and monocytes (see the cell sorting strategy in Figure S7A) with allogenic CD45RA^+^ naive T cells in the presence of a synthetic superantigen. We found that activated DC3s and cDC2s, unlike monocytes, triggered proliferative expansion and effector differentiation in CD4^+^ and CD8^+^ T cells probed by CD45RO acquisition (Figure S7B). The same results were also obtained using *in-vitro-*generated DC3s (Figure S7C). In contrast to monocytes, activated DC3s and cDC2s induced interferon γ (IFN-γ)- and TNF-α-producing CD4^+^ and CD8^+^ T cells but not IL- 17A (Figure 7A; Figure S7D). We found that DC3s had a specific ability to efficiently trigger CD103 expression in CD8^+^ T cells (Figure 7B), even without the presence of superantigen (Figure S7E). CD103 expression is a hallmark of tissue-resident memory T (T_RM_) cells because of its interaction with E-cadherin (Mueller and Mackay, 2016). Mechanistically, multiple factors, including transforming growth factor β (TGF-β), have been proposed to induce CD103 expression on T cells (Mueller and Mackay, 2016; Rihs et al., 1996; Yu et al., 2013). Here we showed that anti-TGF-β neutralizing antibodies, but not others, blocked the expression of CD103 on CD8^+^ T cells (Figure 7C; Figure S7F).

We next wondered whether CD103^+^ T cells aligned with *bona fide* T_RM_ cells isolated from human tissue. To this end, we evaluated the gene expression profile of CD103^−^ and CD103^+^ CD8^+^ T cells obtained after coculture of naive T cells with activated DC3s (Figure S7G). Using the GSEA methodology (Subramanian et al., 2005), we found that the signatures obtained for breast cancer or lung tissue CD103^+^ T_RM_ cells (Hombrink et al., 2016; Kumar et al., 2017; Savas et al., 2018) were enriched in CD103^+^ T cells compared with CD103^−^ T cells induced by DC3s (Figure 7D). DEG analysis revealed that human T_RM_ cell markers such as *NUSAP1*, *DUSP4*, *CXCR6*, and *FASLG* (Figure 7E; Hombrink et al., 2016; Kumar et al., 2017; Savas et al., 2018) were upregulated in CD103^+^ compared with CD103^−^ CD8^+^ T cells. In addition, DC3-activated CD103^+^ CD8^+^ T cells expressed core components of the cytotoxic machinery (*PRF1* and *GZMA*), as reported earlier for breast cancer-invading T_RM_ cells (Figure 7E; Savas et al., 2018).

CD8^+^CD103^+^ T_RM_ cell infiltration has a protective prognosis value in breast cancer (Savas et al., 2018; Wang et al., 2016). To test the physiological relevance of DC3-dependent induction of CD8^+^CD103^+^ T cells, we analyzed CD103 and CD69 expression in CD3^+^CD8^+^ T cells from 18 samples of primary luminal breast cancer (Figure 7F). We found that CD8^+^CD103^+^CD69^+^ T cells were present across different cancer stages (Figure 7G). The frequency of CD8^+^CD103^+^CD69^+^ T cells was positively correlated with DC3 infiltration but not with other mononuclear phagocytes (Figure 7H). Despite the statistical significance of this correlation, it is important to underline that this was mainly driven by a subset of the samples. This suggests that further stratification of patients could help improve our understanding of the relationship between T_RM_ cells and DC3s in breast cancer.

Together, our results anchor DC3 function within the DC lineage; DC3s, just like cDC2s but unlike monocytes, are competent for priming and polarization of CD45RA^+^ naive T cells. In addition, we define induction of *bona fide* T_RM_ cell-like CD103^+^CD8^+^ T cells (Yu et al., 2013) as a specific privilege of DC3s but not cDC2s.

## Discussion

Using scRNA-seq and high-dimensional flow cytometry, we provided evidence that DC3s represent a DC subset that can be separated and isolated from other DC subtypes. Regarding gene expression, our results are largely congruent with findings reported in previous studies (Dutertre et al., 2019; Villani et al., 2017). In addition, we provide a robust flow cytometry strategy to identify and purify DC3s by taking in account CD14^+^ monocytes and cDC2s. In agreement with previous studies, our results challenge the widely accepted notion that CD14 is a specific marker for monocytes (Dutertre et al., 2019; Villani et al., 2017). Indeed, we clearly demonstrate that a large fraction of DC3s expresses cell surface CD14. We identified CD88 (encoded by the *C5AR1* gene) as a proper monocyte marker, enabling prospective purification of monocytes devoid of CD14^+^CD1c^+^ DC3s. We found that *ex vivo* GM-CSF cultures of pure CD88^+^CD14^+^ monocytes did not give rise to CD88^-^CD14^+^CD1c^+^ iDCs. Therefore, our findings provide an incentive to carefully revisit the prevailing notion that CD14^+^CD1c^+^ iDCs arise exclusively from monocytes *in vivo*.

From the DC perspective, we refined the strategy to analyze the functional heterogeneity of CD1c^+^ DCs, including cDC2s and DC3s. Indeed, previous studies have reported heterogeneous expression of CD5 in CD1c^+^ DCs (Dutertre et al., 2019; Yin et al., 2017). However, our findings highlight that CD5^−^CD1c^+^ DCs contain CD163^−^ cDC2s and CD163^+^ DC3s, which are transcriptionally distinct. Overall, our scRNA-seq and bulk RNA-seq analyses cluster together CD5^+^ and CD5^−^ cDC2s. However, the developmental relationship between CD5^−^ cDC2s, CD5^+^ cDC2s, and AS-DCs remains to be clarified (Dutertre et al., 2019; See et al., 2017; Villani et al., 2017).

Because of CD1c expression and their relative similarity to cDC2s, DC3s have been embedded in the group of cDCs. Here we have provided substantial evidence arguing against this notion. Indeed, a conservative definition of cDCs includes (1) dependence on the FLT3L growth factor (Breton et al., 2015; Guermonprez et al., 2013, 2019; McKenna et al., 2000; Pulendran et al., 1998; Waskow et al., 2008) and (2) reliance on the CDP and pre-cDC developmental pathway (Breton et al., 2015, 2016; Guermonprez et al., 2019; Lee et al., 2015b; Naik et al., 2007; Onai et al., 2007). Here we have shown that DC3s do not meet any of these criteria. First, FLT3L alone was poorly active in stimulating the production of DC3s from CD34^+^ HSPCs in a controlled setting *in vitro*. Conversely, GM-CSF drives the commitment of DC3s under the same conditions. Moreover, we have shown that CDP did not give rise to DC3s although they are competent to generate cDC1s and cDC2s (Lee et al., 2015b). Taking in account the heterogeneity of defined progenitor populations (Paul et al., 2015), we developed single-cell cultures enabling analysis of multiple lineage populations. Our single-cell cultures demonstrated that DC3s develop from CLEC12A^+^ DC3-restricted progenitors. In addition, the most frequent progenies differentiating along with DC3s from a bi-potent progenitor were cDCs and macrophages. This strongly suggest that DC3 progenitors diverge downstream of the MDP stage (Fogg et al., 2006; Lee et al., 2015a). Further studies will define more precisely the cell surface and molecular phenotype of the DC3-restricted progenitor. In support of a distinct regulation of cDC2s and DC3s, we have shown that cDC2 infiltration in breast cancer is correlated with cDC1s but not DC3s. Also, Dutertre et al. (2019) have shown recently that DC3s, but not cDC2s, expand in the blood of systemic lupus erythematosus patients (Dutertre et al., 2019). Further delineation of the inflammatory cues and transcription factors underpinning the development of the DC3 lineage is needed.

Identification of DC3 as a cellular entity arising from a specific lineage brings forward the question of their specific immune function. We have shown that activated DC3s, just as cDC2s but unlike monocytes, secrete high amounts of T cell-polarizing cytokines (IL-12p70 and IL-23) and T cell-attracting chemokines (CXCL9, CXCL10, CXCL11, and CCL5). In addition, DC3s also secrete other cytokines (IL-10, IL-6, and TNF-α), some of which were poorly secreted by cDC2s (e.g., TNF-α). DC3s are polyvalent phagocytes with a cytokine pattern encompassing T cell and inflammation cues.

A quintessential defining feature of DCs is their capacity to activate naive T cells. For instance, infiltration of CD1c^+^ DCs is associated with priming of T cell effectors when regulatory T (Treg) cell infiltration is low or Treg cell-mediated suppression is alleviated by checkpoint blockade (Binnewies et al., 2019). However, even when the heterogeneity of CD1c^+^ cells is appreciated, including recruitment of CD14^+^CD1c^+^ DCs in tumor-draining lymph nodes (Binnewies et al., 2019), little is known about the function of CD1c^+^ subtypes. Therefore, we asked whether the functions attributed to CD1c^+^ DCs would be carried out by cDC2s and/or DC3s. We found that DC3s, like cDC2s but unlike monocytes, primed and drove robust activation of naive T cells into IFN-γ- and TNF-α-secreting polyfunctional effectors (Acosta-Rodriguez et al., 2007; Leal Rojas et al., 2017; Napolitani et al., 2005; Nizzoli et al., 2013; Schlitzer et al., 2013; Yin et al., 2017).

αE integrin (CD103) is a key marker of T_RM_ cells through its interaction with E-cadherin, participating in retention of T cells at epithelial and mucosal sites (Mueller and Mackay, 2016). Yu et al. (2013) have identified a feature of total CD1c^+^ DCs in their ability to drive acquisition of CD103 in CD8^+^ T cells. Here we have shown that DC3s, but not cDC2s, induced expression of CD103 on CD8^+^ and CD4^+^ T cells. In addition, we have shown that CD103^+^ T cells primed by DC3s *ex vivo* align with *bona fide* T_RM_ cells isolated from lung or breast cancer (Hombrink et al., 2016; Kumar et al., 2017; Savas et al., 2018). This finding is consistent with the existence of early imprinting of the T_RM_ cell program at the level of T cell priming by DC3s. This notion is supported by a recent *in vivo* study in a mouse model, evidencing early imprinting of the T_RM_ cell program during T cell priming in the lymph nodes by αV integrin-expressing migratory DCs (Mani et al., 2019). Further supporting this view, we found that (1) DC3s upregulated CCR7 when activated by TLR agonists, potentially acting as migratory DCs, and (2) blocking studies indicated that TGF-β was required to prime CD103^+^ T cells. This role of priming in lymph nodes does not exclude that tumor-infiltrating DC3s might also provide TGF-β and other signals important for maintenance of T_RM_ cells, as demonstrated in a mouse model (Mani et al., 2019). In support of this view, we found that infiltration of DC3s was selectively associated with the abundance of CD8^+^CD103^+^ T cells in luminal breast cancer primary tumors. Further studies are needed to delineate the full molecular mechanisms of CD103 induction in T cells by DC3s and their physiological *in vivo* relevance during immune responses. Indeed, CD103^+^CD8^+^ T cells are a protective biomarker in triple-negative breast cancer (Savas et al., 2018; Wang et al., 2016), and lung CD103^+^CD8^+^ T cells are a hallmark of protective immunity afforded by influenza vaccination (Yu et al., 2013). This underlines the potential of DC3s to regulate tissue immunity and defines them as targets for vaccines and immunotherapeutic interventions.

## Limitations of Study

We identify DC3s as CD88^-^CD1c^+^CD163^+^CD14^+/-^. Further studies will be needed to determine if this phenotype corresponds to all CD1c^+^CD14^+^ cells that had been reported in various inflammatory settings.

We showed that DC3 differentiation is driven by GM-CSF in humanized mouse metastatic lung model. However, human circulating PBMCs were used in these experiments, preventing the assessment of GM-CSF impact on bone marrow DC3 progenitors *in vivo*. Hence, we cannot exclude a role of GM-CSF on DC3s survival instead of differentiation. In addition, we showed that GM-CSF drives DC3 differentiation from CD34^+^ umbilical cord HSPCs *in vitro*. This finding does not exclude that other growth factors control DC3 development *in vivo*.

We aligned CD1c^+^CD14^+^ cells infiltrating breast tumor-draining lymph node with blood DC3s compared to cDC2s and monocytes. Due to limited availability of healthy human lymph nodes, we did not investigate if CD1c^+^CD14^+^ cells are infiltrating secondary lymphoid organs in homeostatic conditions. Blood DC3s upregulate CCR7 upon TLR stimulation suggesting they have a migratory potential. However, we did not investigate whether lymph nodes DC3s originate from non-lymphoid peripheral tissues via lymphatic vessels or directly from blood.

A defining feature of cDCs lies in their ability to prime naïve T cells. We showed that DC3s can induce the proliferation of naïve T cell in a non-autologous priming context, *i.e.* the mixed leukocyte reaction. The capability of DC3s to uptake, process and present antigens by MHCI and MHCII molecules remains to be addressed.

Our *in vitro* experiments showed that ***i***) DC3s induce efficiently T_RM_ differentiation from naïve CD8^+^T cells and, ***ii***) DC3 infiltration correlates with T_RM_ abundance *in vivo*. However, this does not provide a direct evidence that DC3s control T_RM_ specification within tumor draining lymph nodes where they are likely to interact with naïve T cells. Indeed, we cannot rule out that, *in vivo*, DC3s act selectively in the tissue to maintain T_RM_ populations primed in lymph nodes by a different DC subtype. Further studies will be needed to address how DC3s control CD8^+^ T_RM_ populations *in vivo*.

## STAR★Methods

### Key Resources Table

**Table:** 

REAGENT or RESOURCE	SOURCE	IDENTIFIER
**Antibodies**		

Biotin anti-human CD335 (NKp46) antibody (clone 9E2)	BioLegend	Cat#331906; RRID: AB_1027671
Biotin anti-human CD3 antibody (clone OKT3)	BioLegend	Cat# 317320; RRID: AB_10916519
Biotin anti-human CD19 antibody (clone HIB19)	BioLegend	Cat# 302204; RRID: AB_314234
Biotin anti-human CD20 antibody (clone 2H7)	BioLegend	Cat# 302350; RRID: AB_2565524
Biotin anti-human CD56 (NCAM) antibody (clone HCD56)	BioLegend	Cat# 318320; RRID: AB_893390
Biotin anti-human CD203c (E-NPP3) antibody (clone NP4D6)	BioLegend	Cat# 324604; RRID: AB_756042
Biotin anti-human CD66b antibody (clone G10F5)	BioLegend	Cat# 305120; RRID: AB_2566608
Brilliant Violet 421 anti-human CD16 antibody (clone 3G8)	BioLegend	Cat# 302038; RRID: AB_2561578
Brilliant Violet 510 anti-human CD45RA antibody (clone HI100)	BioLegend	Cat# 304142; RRID: AB_2561947
Brilliant Violet 510 anti-human HLA-DR antibody (clone L243)	BioLegend	Cat# 307646; RRID: AB_2561948
Brilliant Violet 510 anti-human CD15 (SSEA-1) antibody (clone W6D3)	BioLegend	Cat# 323028; RRID: AB_2563400
FITC anti-human CD163 antibody (clone GHI/61)	BioLegend	Cat# 333618; RRID: AB_2563094
Brilliant Violet 711 anti-human CD163 antibody (clone GHI/61)	BioLegend	Cat# 333630; RRID: AB_2650972
Brilliant Violet 421 anti-human CD163 antibody (clone GHI/61)	BioLegend	Cat# 333612; RRID:AB_2562463
Brilliant Violet 785 anti-human CD14 antibody (clone M5E2)	BioLegend	Cat# 301840; RRID: AB_2563425
PE/Cy7 anti-human CD14 antibody (clone HCD14)	BioLegend	Cat# 325618; RRID: AB_830691
FITC anti-human CD14 antibody (clone M5E2)	BioLegend	Cat# 301804; RRID: AB_314186)
Brilliant Violet 785 anti-human CD3 antibody (clone UCHT1)	BioLegend	Cat# 300472; RRID: AB_2687178
APC anti-human CD34 antibody (clone 561)	BioLegend	Cat# 343608; RRID: AB_2228972
FITC anti-human CD34 antibody (clone 561)	BioLegend	Cat# 343604; RRID: AB_1732005
PerCP/Cyanine5.5 anti-human CD303 (BDCA-2) antibody (clone 201A)	BioLegend	Cat# 354210; RRID: AB_11219604
PerCP/Cyanine5.5 anti-human CD141 (Thrombomodulin) antibody (clone M80)	BioLegend	Cat# 344112; RRID: AB_2561625
PerCP/Cyanine5.5 anti-human CD371 (CLEC12A) antibody (clone 50C1)	BioLegend	Cat# 353612; RRID: AB_2565544
PE anti-human CD370 (CLEC9A/DNGR1) antibody (clone 8F9)	BioLegend	Cat# 353804; RRID: AB_10965546
PE anti-human CD5 antibody (clone L17F12)	BioLegend	Cat# 364014; RRID: AB_2565284
PE anti-human CD116 antibody (clone 4H1)	BioLegend	Cat# 305908; RRID: AB_2085686
PE anti-human CD206 (MMR) antibody (clone 15-2)	BioLegend	Cat# 321106; RRID: AB_571911
PE anti-human CD88 (C5aR) antibody (clone S5/1)	BioLegend	Cat# 344304; RRID: AB_2067175
PE/Dazzle 594 anti-human CD123 antibody (clone 6H6)	BioLegend	Cat# 306034; RRID: AB_2566450
PerCP/Cyanine5.5 anti-human CD123 antibody (clone 6H6)	BioLegend	Cat# 306016; RRID: AB_2264693
FITC anti-human CD1c antibody (clone L161)	BioLegend	Cat# 331518; RRID: AB_2073403
PE/Cy7 anti-human CD1c antibody (clone L161)	BioLegend	Cat# 331516; RRID: AB_2275574
APC anti-human CD272 (BTLA) antibody (clone MIH26)	BioLegend	Cat# 344510; RRID: AB_10613101
APC anti-human CD64 antibody (clone 10.1)	BioLegend	Cat# 305014; RRID: AB_1595428
APC/Cyanine7 anti-human CD45 antibody (clone HI30)	BioLegend	Cat# 304014; RRID: AB_314402
APC/Cy7 Streptavidin	BioLegend	Cat# 405208
Brilliant Violet 785 anti-human CD95 (Fas) antibody (clone DX2)	BioLegend	Cat# 305646; RRID: AB_2629742
Brilliant Violet 711 anti-human CD8 antibody (clone SK1)	BioLegend	Cat# 344734; RRID: AB_2565243
Brilliant Violet 605 anti-human CD45 antibody (clone HI30)	BioLegend	Cat# 304042; RRID: AB_2562106
Brilliant Violet 650 anti-human CD45RO antibody (clone UCHL1)	BioLegend	Cat# 304232; RRID: AB_2563462
Brilliant Violet 421 anti-human CD103 (Integrin E) antibody (clone Ber-ACT8)	BioLegend	Cat# 350214; RRID: AB_2563514
Brilliant Violet 785 anti-human CD103 (Integrin E) antibody (clone Ber-ACT8)	BioLegend	Cat# 350230; RRID: AB_2734364
FITC anti-human CD223 (LAG-3) antibody (clone 7H2C65)	BioLegend	Cat# 369210; RRID: AB_2716129
FITC anti-human CD3 antibody (clone UCHT1)	BioLegend	Cat# 300406; RRID: AB_314060
PE/Cy7 anti-human CD183 (CXCR3) antibody (clone G025H7)	BioLegend	Cat# 353720; RRID: AB_11219383
PE/Dazzle 594 anti-human CD28 antibody (clone CD28.2)	BioLegend	Cat# 302942; RRID: AB_2564235
PE anti-human CD197 (CCR7) antibody (clone G043H7)	BioLegend	Cat# 353204; RRID: AB_10913813
APC/Cyanine7 anti-human CD4 antibody (clone SK3)	BioLegend	Cat# 344616; RRID: AB_2028483
Alexa Fluor® 700 anti-human CD127 (IL-7Ra) antibody (clone A019D5)	BioLegend	Cat# 351344; RRID: AB_2566200
APC anti-human CD279 (PD-1) antibody (clone EH12.2H7)	BioLegend	Cat# 329908; RRID: AB_940475
PE anti-human CD115 antibody (clone 9-4D2-1D4)	BioLegend	Cat# 347310; RRID: AB_2565491
FITC anti-human CD116 antibody (clone 4H1)	BioLegend	Cat# 305906; RRID: AB_2085687
FITC anti-human CD86 antibody (clone BU63)	BioLegend	Cat# 374204; RRID: AB_2721574
Human Axl PE antibody (clone 108724)	R&D	Cat# FAB154P; no RRID
PerCP/Cyanine5.5 anti-mouse CD45 antibody (clone 30-F11)	BioLegend	Cat# 103132; RRID: AB_893340
APC-eFluor 780 anti-human HLA-DR antibody (clone LN3)	eBioscience	Cat# 47-9956; RRID:AB_1963603
PE/Cy7 anti-human CD11c antibody (clone Bu15)	Biolegend	Cat# 337216; RRID:AB_2129790
FITC anti-human CD14 antibody (clone 61D3)	eBioscience	Cat# 11-0149; RRID:AB_464951
PerCP-eFluor 710 anti-human CD1c antibody (clone L161)	eBioscience	Cat# 46-0015; RRID:AB_10548936
PE anti-human CD304 antibody (clone AD5-17F6)	Miltenyi	Cat# 130-113-517; RRID:AB_2751124
PE-Cy5 Mouse anti-Human CD1a antibody (clone HI149)	BD	Cat# 555808; RRID:AB_396142
Alexa Fluor® 647 anti-human CD206 (MMR) antibody (clone 15-2)	Biolegend	Cat# 321116; RRID:AB_571881
VioBlue anti-human CD141 antibody (clone AD5-14H12)	Miltenyi	Cat# 130-113-882; RRID:AB_2726374
Purified anti-human CD45 antibody (clone HI30)	BioLegend	Cat# 304002; RRID: AB_314390
Purified anti-human TGF 1-2-3 antibody (clone 1d11)	R&D	Cat# MAB1835; RRID:AB_357931
Purified anti-human TNFα antibody (clone 1825)	R&D	Cat# MAB210; RRID:AB_2240620
Purified anti-human IL-15 antibody (clone 34593)	R&D	Cat# MAB247; RRID:AB_2124578
Purified anti-human Lymphotoxin α antibody (clone 5802)	R&D	Cat# MAB211; RRID:AB_2138622
Purified polyclonal anti-human OX40L antibody	R&D	Cat# AF1236; RRID:AB_354686
Purified polyclonal anti-human IL-33 antibody	R&D	Cat# AF3625; RRID:AB_1151900
Purified anti-human TNFSF8 antibody (clone 116614)	R&D	Cat# MAB1028; RRID:AB_2303710
Purified Mouse IgG1 (clone 11711)	R&D	Cat# MAB002; RRID:AB_357344
Purified polyclonal Goat IgG	R&D	Cat# AB-108-C; RRID:AB_354267
Purified Mouse IgG2b (clone 20116)	R&D	Cat# MAB004; RRID:AB_357346

**Bacterial and Virus Strains**		

pMX-IRES-GFP	Cell biolabs	Cat# RTV-0133
pMX-huFLT3L-IRES-GFP	This paper	N/A
pMX-huGMCSF-IRES-GFP	This paper	N/A

**Biological Samples**		

Leukocyte cones for bulk RNaseq	NHS	N/A
Leukocytes cones for functionnal experiments	NHS	N/A
umbilical cord-blood for *in vitro* diffentiation cells	Anthony Nolan Cell Therapy Centre	N/A
PBMC from whole blood for blood scRNaseq	Institut Curie Hospital (Paris, France)	N/A

**Chemicals, Peptides, and Recombinant Proteins**		

Liberase TL	Sigma	Cat# 5401020001
Collagenase D	Sigma	Cat# 11088866001
DNase I	Sigma	Cat# 10104159001
Dispase	Sigma	Cat# D4693
CD34 MicroBead Kit UltraPure, human	Miltenyi	Cat# 130-100-453
Accucheck counting beads	Thermo Fischer	Cat# PCB100
Matrigel®	BD	Cat# 354230
Poly I:C	InvivoGen	Cat# 31852-29-6
R848	InvivoGen	Cat# 144875-48-9
lipopolysaccharide (LPS)	Sigma-Aldrich	Cat# L2630
Cell Trace Violet (CTV)	Thermo Fischer	Cat# C34557
Naive Pan T cells isolation Kit	Miltenyi	Cat# 130-097-095
Cytostim	Miltenyi	Cat# 130-092-172
Cytofix/ cytoperm Kit	BD Bioscience	Cat# 554714

**Critical Commercial Assays**		

Human Flt-3 Ligand/FLT3L Quantikine ELISA Kit	R&D	Cat# DFK00
ELISA MAX Deluxe Set Human GM-CSF	Biolegend	Cat# 432004
Human Proinflammatory Chemokine Panel (13-plex)	Biolegend	Cat# 740003
Human Macrophage/Microglia Panel (13-plex)	Biolegend	Cat# 740503

**Deposited Data**		

RNaseq data raw reads and processed data	This paper	GEO: SuperSeries GSE151095

**Experimental Models: Cell Lines**		

B16_Control	This paper	N/A
B16_human FLT3L	This paper	N/A
B16_human GM-CSF	This paper	N/A
MS5_FLT3L	Anselmi et al., 2020	N/A
MS5_GM-CSF	Anselmi et al., 2020	N/A
MS5_FS12	Anselmi et al., 2020	N/A

**Experimental Models: Organisms/Strains**		

mouse: NOD.Cg-Prkdcscid Il2rgtm1Wjl/SzJ	The Jakson Laboratory	Stock#005557

**Software and Algorithms**		

Genomics Suite	Partek	N/A
FlowJo V10	BD	https://www.flowjo.com
GraphPad Prism 8	GraphPad Software	https://www.graphpad.com
R4.4	The R Foundation	https://www.r-project.org
Fiji	open source	https://imagej.net/Fiji

### Resource Availability

#### Lead Contact

Additional information and request for resources and reagents should be directed to and will be made available by the Lead Contact, Pierre Guermonprez (pierre.guermonprez@kcl.ac.uk).

#### Materials Availability

The reagents generated in this study will be made available on request, but we may require a payment and/or a completed Materials Transfer Agreement if there is potential for commercial application.

#### Data and Code Availability

The scRNA-seq and the bulk RNA-seq datasets are deposited in the Genome Expression Omnibus under the SuperSeries accession numbers GSE151095.

### Experimental Model and Subject Details

#### Human umbilical cord and adult blood

Human umbilical cord blood units were obtained from Anthony Nolan Cell Therapy Centre (ANCTC). Leukophoretic adult blood (buffy coats or leukocyte cones) were obtained from healthy volunteers through NHS.

#### Clinical samples

Tumor-invaded lymph nodes (tdLN) and primary tumors were collected from luminal breast cancer submitted to surgical resection at the Institut Curie Hospital (Paris, France), in accordance with institutional ethical guidelines. Patients’ clinical and pathologic characteristics are summarized in Table S2.

#### Mice

NSG (NOD.Cg-*Prkdc*^*scid*^ *Il2rg*^*tm1Wjl*^/SzJ) mice were bred and maintained in specific pathogen-free animal facility in accordance with institutional KCL guidelines. All procedures involving animals were conducted according to requirements of UK Animals (Scientific Procedures) Act 1986.

### Method Details

#### Human blood and CD34^+^ progenitors

Peripheral blood mononuclear cells (PMBCs) were obtained by gradient centrifugation using Ficoll-Paque (GE Healthcare). Progenitor cells were enriched using CD34^+^ microbead isolation kit (Miltenyi).

#### Tumor cell lines

B16_CTRL, B16_huGM-CSF and B16_huFLT3L were generated by retroviral transduction of B16-F10 (C57BL/6 melanoma cell line) with an empty pMX-IRES-GFP vector or coding for human GM-CSF and human Flt3L respectively. Tumor cell lines were cultured in RPMI 1640 medium (Thermo Fisher) supplemented with 10% fetal bovine serum (FBS) (Thermo Fisher), penicillin-streptomycin (Thermo Fisher) and b-mercaptoethanol (Thermo Fisher) (complete RPMI) and maintained at 37°C and 5% CO_2_.

#### Metastasis model

Engineered B16-F10 cells were counted and resuspended in RPMI 1640 medium. NSG mice (8-12 weeks, males and females) were injected intravenously with 10^6^ B16-F10 at day 0. 10^8^ human blood PBMCs were injected intravenously at day 7 and 8. Mice were culled at day 9 and lungs were harvested.

#### Histology

Mouse lungs were fixed with 1% PFA (Alfa Aesar) for 1hr at 4°C, washed and incubated in 34% sucrose solution (Sigma-Aldrich) overnight at 4°C. Lungs were embedded in Cryomatrix (Thermo Fischer) and frozen for cryostat sectioning (9 μm-thick). Sections were permeabilized using 0.5% saponin (Sigma-Aldrich), 2% BSA (Sigma-Aldrich), 1% FBS (Life Technologies) for 30 minutes at room temperature. Sections were labeled overnight at 4°C with mouse anti-human purified CD45 (HI30, Biolegend) followed by incubation for 1hr at room temperature with goat anti-mouse Cy3 (Jackson laboratory). All sections were labeled with Hoechst (Molecular Probes, Thermo Fisher) for nuclei staining 5 minutes at room temperature and mounted with Prolong diamond (Thermo scientific). Slides were imaged using a SP5 (Leica) and analyzed with Fiji software.

#### Preparation of cell suspensions from lung mouse

Mouse lungs were harvested and transferred to 3ml digestion buffer (Hank’s Balanced Salt Solution (HBSS) with calcium and magnesium (Thermo Fisher) and with 0.1 mg/ml of Liberase TL (Roche) and 0.02μg/ml DNase I (Thermo Fisher). Lungs were dissociated using gentleMACS Octo Dissociator (Miltenyi) and incubated at 37°C for 45 minutes. The cell suspension was passed through a cell strainer (70μm, Corning) and red blood cells were lysed using ACK lysing buffer (Thermo Fischer). The absolute number of cells in the resulting cell suspension was assessed using AccuCheck Counting Beads (Thermo Fisher) on BD FACSCanto II (BD Biosciences).

#### Stromal cell line maintenance

Mouse bone marrow-derived MS5 stromal cells engineered to express human membrane bound FLT3L alone (MS5_FL) or in combination with SCF and soluble CXCL12 (MS5_FS12) or human GM-CSF alone (MS5_GM) generated as previously described (Anselmi et al., 2020). Stomal cell lines were cultured in IMDM medium supplemented with 10% heat-inactivated FBS, penicillin/streptomycin, 50 μM β-mercaptoethanol (complete IMDM) and maintained at 37°C and 5% CO_2_.

#### *In vitro* differentiation from CD34^+^ progenitors or blood phagocytes

For *in vitro* co-culture experiments with stromal cells, MS5_FL, MS5_FS12 and MS5_GM feeders were seeded at 10^4^ cells/well density in a 96-well plate (Thermo Fischer) and maintained overnight at 37°C and 5% CO_2_.

Total enriched-CD34^+^ progenitors and flow cytrometry-sorted progenitor subsets (GMDP, CDP and cMoP) were plated on top of stromal cells, supplemented or not with 100ng/ml of recombinant human GM-CSF (Peprotech), for 14 days and 7 days, respectively. Blood phagocytes subsets were flow cytometry-sorted and plated on MS5_GM for 2 days.

For bulk RNA sequencing analysis, total enriched-CD34^+^ progenitors were plated on top of MS5_FS12 feeders and with or without 100ng/ml of recombinant human GM-CSF (Peprotech). On day 5 and 10 of differentiation, half the medium was replaced with fresh complete IMDM or complete IMDM containing 100ng/ml of recombinant human GM-CSF. Cells were collected at day 15.

All cells were collected with a solution of phosphate buffered saline (PBS) (GIBCO) 5mM EDTA (Thermo Fisher) at 4°C for 10min.

#### *In vivo* conversion assay

PBMCs from healthy donors were stained and sorted as described previously. Blood cDC2 (10^5^) and monocytes (3 × 10^5^) were flow cytometry-sorted and injected subcutaneously along with B16_huGM (10^5^) in 200 μL of ice-cold Matrigel**®** (BD Biosciences). Mice were sacrificed at day 4 by cervical dislocation and Matrigel**®** plugs were collected. Subcutaneous Matrigel**®** plugs were recovered, cut in pieces and incubated in HBSS (Life Technologies) 1% FBS, 0.37 U/ml Collagenase D (Roche), 10 μg/ml DNaseI (Roche) and 1 mg/ml Dispase (Sigma-Aldrich) for 30 minutes at 37°C. After digestion, plugs were smashed on a 70 μm strainer (Corning) and cells were collected and resuspended in flow cytometry buffer for flow cytometry analysis.

#### Flow cytometry analysis

Cells were stained in flow cytometry buffer (PBS 5mM EDTA 1% BSA) according to antibody panels (Key Resources Table) for 30 min. Dead cells were stained using DAPI or Live/Dead Blue staining (Thermo Fischer). Lineage (Lin) included CD3, CD19, CD20, NKp46, CD56, CD203c and CD66b, all conjugated with biotin. Multiparameter analysis was performed on LSRFortessa and Symphony (BD Biosciences) flow cytometers and analyzed using FlowJo software (Tree Star). The flow cytometry-sort was performed on BD FACS Aria II or BD FACS Aria Fusion at the Biomedical Research Centre (BRC) Flow Core Facility (Guy’s and St Thomas’ NHS Foundation Trust and King’s College London).

Unsupervised clustering of flow cytometry data (Figure 1F) was performed using Self-Organizing Map clustering algorithm FlowSOM (Cytofkit R package). Data was then extracted as FCS files and further analyzed in FlowJo software.

#### Bulk RNA sequencing

For bulk sequencing of *in vitro* differentiated subsets, up to 100 flow cytometry-sorted cells from three individual donors were collected directly in Lysis buffer (Takara Clontech, Cat# 635015) containing RNase inhibitors. RNaseq libraries were prepared on the contactless liquid handling system Labcyte Echo 525 (Labcyte Inc). In brief, ERCC was added to each sample and first strand full length cDNA was generated with a modified protocol of the SMARTseq v4 Ultra Low Input RNA Kit (Takara Clontech, Cat# 634891) using poly dT primers and a template switching oligo. Full length cDNA was amplified using SeqAmp DNA Polymerase (Takara Clontech, Cat# 638509). 12 ng of amplified cDNA from each sample was used to generate non-stranded RNA libraries using a modified protocol of the Ovation Ultralow System V2 1-96 kit (NuGEN, Cat# 0347-A01). In brief, amplified cDNA was fragmented through sonication on Covaris E220 (Covaris Inc), repaired and polished followed by ligation of indexed adapters. Adaptor ligated cDNA were pooled before final amplification to add flow cell primers. Libraries were sequenced on HiSeq2500 (Illumina Cambridge) for 100 cycles PE in Rapid mode.

#### Bulk RNA sequencing data processing

The raw sequencing data was initially processed using open source, web-based platform Galaxy (version 18.05.rc1) (https://usegalaxy.org). Reads were filtered for quality with more than 80% of the sequence having quality score > 33 using FastQC tool. Mapping against reference genome was performed with Hisat2 to the hg38 human genome. Adaptor sequences were detected automatically with TrimGalore!. Reads under 20bp were discarded. All processed sequencing files were imported in Partek® Genomics Suite software®, version 7.0©; 2017 (PGS), where they were processed further.

#### Primary data analysis and visualization

mRNA was quantified using PGS built in RNA-seq workflow. Normalization method used was Reads Per Kilobase per Million mapped reads (RPKM) and mRNA was quantified against RefSeq Transcripts 2018-11-20 database. Hierarchal clustering on average expression within the group was performed on all identified protein coding genes (19791 genes). Based on the CD14^-^CD1c^+^CD206^+/−^ two blue cluster similarity, they were both considered as cDC2-like cells and their datasets were merged for further analysis. The same was done for the two macrophage-like cells (gray and brown). Differentially expressed genes (Fold-Change ≤ −2 or ≥ 2 and p value < 0.05) were determined using one-way ANOVA in all pairwise comparisons with three donors grouped and visualized as Volcano plots in PGS. Individual samples were visualized via principal component analysis (PCA) using 500 most variable genes, which were determined based on median absolute deviation (MAD). The expression patterns of selected gene lists were displayed in the form of heatmaps, where rows and/or columns were ordered based on hierarchical clustering using Euclidean distance and average linkage in Morpheus (Broad Institute; https://software.broadinstitute.org/morpheus/). A few gene names are depicted next to the heatmap.

#### Single-cell RNA sequencing

PBMCs from three healthy donors or human CD45^+^ cells isolated from metastatic mouse lungs were isolated as previously described. Sorted cells from each donor were pooled together and CD1c^+^ and CD14^+^ cells were mixed at 80:20 ratio. 3 × 10^3^ cells from the resulting cell suspension were partitioned into an emulsion of nanoliter-sized droplets using a 10X Genomics Chromium Single Cell Controller and RNA sequencing libraries were constructed using the Chromium Single Cell 3′ Library & Gel Bead Kit v2 (10X Genomics, Cat# PN-120237). Briefly, droplets containing individual cells, reverse transcription reagents and a gel bead were loaded with poly(dT) primers that include a 16 base cell barcode and a 10 base unique molecular index (UMI). Reverse transcription reactions were engaged to generate barcoded full-length cDNA followed by the disruption of emulsions using the recovery agent and cDNA clean up with DynaBeads MyOne Silane Beads (Thermo Fisher Scientific, Cat# 37002D). Bulk cDNA was amplified, and indexed sequencing libraries were constructed using the reagents from the Chromium Single Cell 3′ v2 Reagent Kit. Libraries were sequenced on NovaSeq 6000 Sequencing System (Illumina Cambridge).

#### Single-cell RNaseq data processing and analysis

Cell Ranger (version 2.1.1) (from 10x genomics) was used to process Chromium single cell 3′ v2 RNA-seq output files. First, we generated fastq files for the Read1 for cell barcode and UMI and Read2 for transcript applying cellranger mkfastq (with default parameters). Second, we aligned the Read2 to the human reference genome GRCh38 using STAR (version 2.5.1) with cell ranger count (with default parameters) (https://support.10xgenomics.com/single-cell-gene-expression/software/pipelines/latest/using/mkfastq). Further analysis was performed using Seurat package (version 2.3.4) in R (version 3.4.0) (Butler et al., 2018). Before performed analysis, we applied the following filtering step: only genes expressed in 3 or more cells have been preserved and cells with less 200 unique genes and more than 4000 unique expressed genes were discarded (as they are potentially cells doublets). After filtering step, we used an expression matrix resulting in 14933 genes across 1622 cells (among 1625 cells) for the rest of the analysis. The matrix was normalized using genes expression values for each cell were divided by the total number of transcripts and multiplied by median of UMI counts. Then, these values were natural log-transformed before downstream analysis. For dimensionality reduction analysis, we first identified 3288 genes as highly variable genes across the single cells (cutoff value for dispersion = 0.5; cutoff value for average expression = 0). PCA performed using the variable genes as input and determined 10 PCs as significant PCs. These principal components were used as input for t-Distributed Stochastic Neighbor Embedding (tSNE) (van der Maaten, 2008). We used the shared nearest neighbor (SNN) modularity optimization-based clustering algorithm from the Seurat package (FindClusters function with default parameters) to identify the clusters of cells, following by Clustree analysis (clustree R package, version 0.2.2) by changing the resolution parameters from 0 to 2. Finally, we kept a resolution parameter at 0.8 and defined distinct 9 clusters. After controlling expression of some quality control genes, we excluded clusters E, F, G, H and I as contamination. Finally, we identified 4 relevant clusters. We identified cell specific marker by comparing cells in a specific cluster with cells in all other clusters using FindAllMarkers from Seurat package (MAST; logFC threshold = 0.5; only positive markers). Heatmap, feature plots and violin plots were performed using Seurat package.

#### Signature expression analysis

Single-cell RNA sequencing matrix for clusters A, B, C and D was created with gene signatures from Villani et al. (2017). Signatures were defined as mean expression of discriminative markers for cDC2s and DC3s among lin^-^CD14^-^ cells (cDC2 enriched and DC3 enriched, respectively) and of discriminative markers for CD14^+^ monocytes within lin^-^CD14^+/−^ and/or CD16^+/−^ monocytes (CD14^+^ mono enriched). The average expression of signature genes for each cell was calculated and plotted as a violin plot using R package ggplot2 (version 3.1.0).

#### Gene set enrichment analysis

To statistically evaluate the enrichment of previously reported gene signatures (Gene Sets) (Hombrink et al., 2016; Kumar et al., 2017; Savas et al., 2018; Villani et al., 2017) in our dataset, we used pairwise comparisons using the gene set enrichment analysis (GSEA) (Subramanian et al., 2005) method from the Massachussets Institute of Technology (https://www.broadinstitute.org/gsea). GSEA tests the relative position of a collection of genes (Gene Set) within an independent, ranked dataset (GeneList). Statistical analysis was performed by evaluation of nominal p value and false discovery rate (q value) based on 1,000 random permutations. Results were considered significant when the p value was below 0.05 and when the q value was below 0.25 (false discovery rate below 25%) accordingly to the recommendation from the software developers. For each pairwise comparison, the GSEA output can be represented as a bar code where each bar corresponds to the projection of one Probe Sets of the Gene Sets on the list of all the Probe Sets of the gene chips ranked from those having a high signal in one cell population (represented in red) to those having a high signal in the other cell population (represented in blue). Each bar code can be characterized by two parameters. The normalized enrichment score (NES) represents the number and differential expression intensity of the genes enriched in the corresponding cell subset. The NES is positive if the Gene Set is enriched in the first cell population and negative if it is enriched in the second cell population. The false discovery rate (FDR) statistical value (q) represents the likelihood that the enrichment of the Gene Set represents a false positive finding (for example if q = 0.25, 25% of the Gene Sets found enriched can be false positives). An absolute value of the NES below or around 1 means no enrichment as confirmed with associated q-values above 0.25.

To simultaneously visualize pairwise comparisons of transcriptomes from cord blood-derived DC2s (light and dark blue) DC3s (orange) and macrophages (gray and brown), the BubbleMap module of BubbleGum (Spinelli et al., 2015) was used. Results were considered significant when the p value was below 0.05 and the FDR (false discovery rate, q) value was below 0.25. The BubbleGum was performed using previously published gene signatures of pairwise comparison between DC2s and DC3s and DC3s and CD14^+^ monocytes (Villani et al., 2017).

#### Human tissue processing and cell suspension

Tumor-draining lymph nodes (tdLN) and primary tumors were collected in CO_2_ independent medium (GIBCO; Cat# 18045-054) within few hours after the primary surgery. Tissue were cut into small fragments and submitted to enzymatic digestion using 0.1 mg/ml of Liberase TL (Roche) and 0.1 mg/ml of DNase (Roche) for 30 min. Cells were filtered on 40-μm cell strainer (BD), washed using CO_2_ independent medium (GIBCO; Cat# 18045-054) containing 0.4g/ml of human albumin and resuspended for cell counting.

#### Cell sorting of myeloid subsets from patients’ tdLN for RNA sequencing

After tissue processing, cells obtained from tdLN were submitted to myeloid cells enrichment accordingly to (Durand and Segura, 2016) prior flow cytometry-sorting. In brief, T and B lymphocytes, NK cells, erythrocytes and myelomonocytic cells were depleted using monoclonal antibodies against: CD3, CD19, CD56, CD235a and CD15, respectively. Subsequently, cell suspensions were stained for 30 min with antibody-conjugated as the following: HLA-DR, CD11c, CD14, CD1c, CD304, CD1a, CD206, CD141. Around 1,000 cells of each DC subset were sorted by flow cytometry using BD FACS ARIA II cell sorter, (purity > 98%). Cells were centrifuged and lysed with TCL buffer (QIAGEN) containing 1% of beta-mercaptoethanol before storage at −80°C. RNA were extracted and isolated using the Single Cell RNA purification kit (Norgen, Cat#51800) according to the manufacturer’s instructions and the RNA integrity number was evaluated with an Agilent RNA 6000 pico kit.

#### LEGENDplex™ assay

PBMCs and *in vitro* generated cells were stained and sorted as described previously. In total 3 × 10^5^ of blood cell subsets (cDC2, DC3 and Mono) or 10^5^ *in vitro* generated cells (cDC2-, DC3 and Macro-like) were flow cytometry-sorted and cultured with TLR agonists cocktail containing 25 μg/ml Poly I:C (InvivoGen, Cat# 31852-29-6), 1 μg/ml R848 (InvivoGen, Cat# 144875-48-9) and 10ng/ml LPS (Sigma-Aldrich, Cat# L2630) for 16 h. Culturing supernatants were collected and stored at −20°C until the LEGENDplex™ assay execution day. LEGENDplex Human Macrophage/Microglia Panel (13-plex) with V-bottom Plate (Biolegend Cat# 740503) and Human Proinflammatory Chemokine Panel (13-plex) with V-bottom Plate (Biolegend Cat# 740003) was used according to manufacturer’s instructions. In short, samples and standards were thawed and plated with capture beads and incubated for 2 h. Plate was then washed, and Detection Antibodies were added. After 1 h incubation SA-PE was added and incubated for 30 min. Samples were acquired on BD FACSCanto II. Samples were analyzed using LEGENDplex™ Data Analysis Software.

#### *In vitro* T cell assay

PBMCs and *in vitro* generated cells were stained and flow cytometry-sorted as described previously. 10^4^ mononuclear phagocytes were cultured with TLR agonists cocktail containing 25 μg/ml PolyI:C, 1 μg/ml R848 and 10 ng/ml LPS for 16 h. T cells were isolated from fresh or frozen PBMCs using Naive Pan T Cell Isolation Kit (Miltenyi, Cat# 130-097-095) and 10^5^ cells were plated on top of mononuclear phagocytes in presence of CytoStim (Miltenyi, Cat# 130-092-172), according to manufacturer’s instructions. For mixed leukocyte reaction (MLR) experiments, isolated Naive Pan T cells were labeled with Cell Tracer Violet (CTV) (Thermo Fischer, Cat# C34557) as per manufacturer’s instructions and cultured with flow cytometry-sorted DCs without CytoStim. At day 5, cells were collected and stained for extracellular and intracellular marker expression and analyzed using BD LSRFortessa. For intracellular staining, fixation and permeabilization were performed using BD Cytofix/Cytoperm solution (BD Biosciences, Cat# 554714) according to manufacturer’s instructions.

### Quantification and Statistical Analysis

Statistical analysis was performed using Prism 8.3 (GraphPad Software Inc., USA). When two experimental groups were compared, non-parametric Mann-Whitney test was used. When three or more groups were compared, statistically significant differences between means were determined using the one-way or two-way analysis of variance (ANOVA) method. A p value of less than 0.05 was considered as significant.

## Supplemental Information

Document S1. Figures S1–S7 and Table S2

Table S1. Significant DEGs Obtained from the Pairwise Comparisons Used to Generate the Volcano Plots, Related to Figures 4 and 6 and Figures S1, S3, S5, and S6

Document S2. Article plus Supplemental Information
