# Supplementary material for: Transcriptional and Functional Analysis of CD1c+ Human Dendritic Cells Identifies a CD163+ Subset Priming CD8+CD103+ T Cells
Source: Immunity. 2020 Aug 18;53(2):335–352.e8. doi: 10.1016/j.immuni.2020.06.002 (PMC7445430; doi:10.1016/j.immuni.2020.06.002)
Supplement: Document S1. Figures S1–S7 and Table S2 [file mmc1.pdf]

Immunity, Volume *53*

Supplemental Information

Transcriptional and Functional Analysis of CD1c$^+$ Human Dendritic Cells Identifies a CD163$^+$ Subset Priming CD8$^+$CD103$^+$ T Cells

Pierre Bourdely, Giorgio Anselmi, Kristine Vaivode, Rodrigo Nalio Ramos, Yoann Missolo-Koussou, Sofia Hidalgo, Jimena Tosselo, Nicolas Nuñez, Wilfrid Richer, Anne Vincent-Salomon, Alka Saxena, Kristie Wood, Alvaro Lladser, Eliane Piaggio, Julie Helft, and Pierre Guermonprez

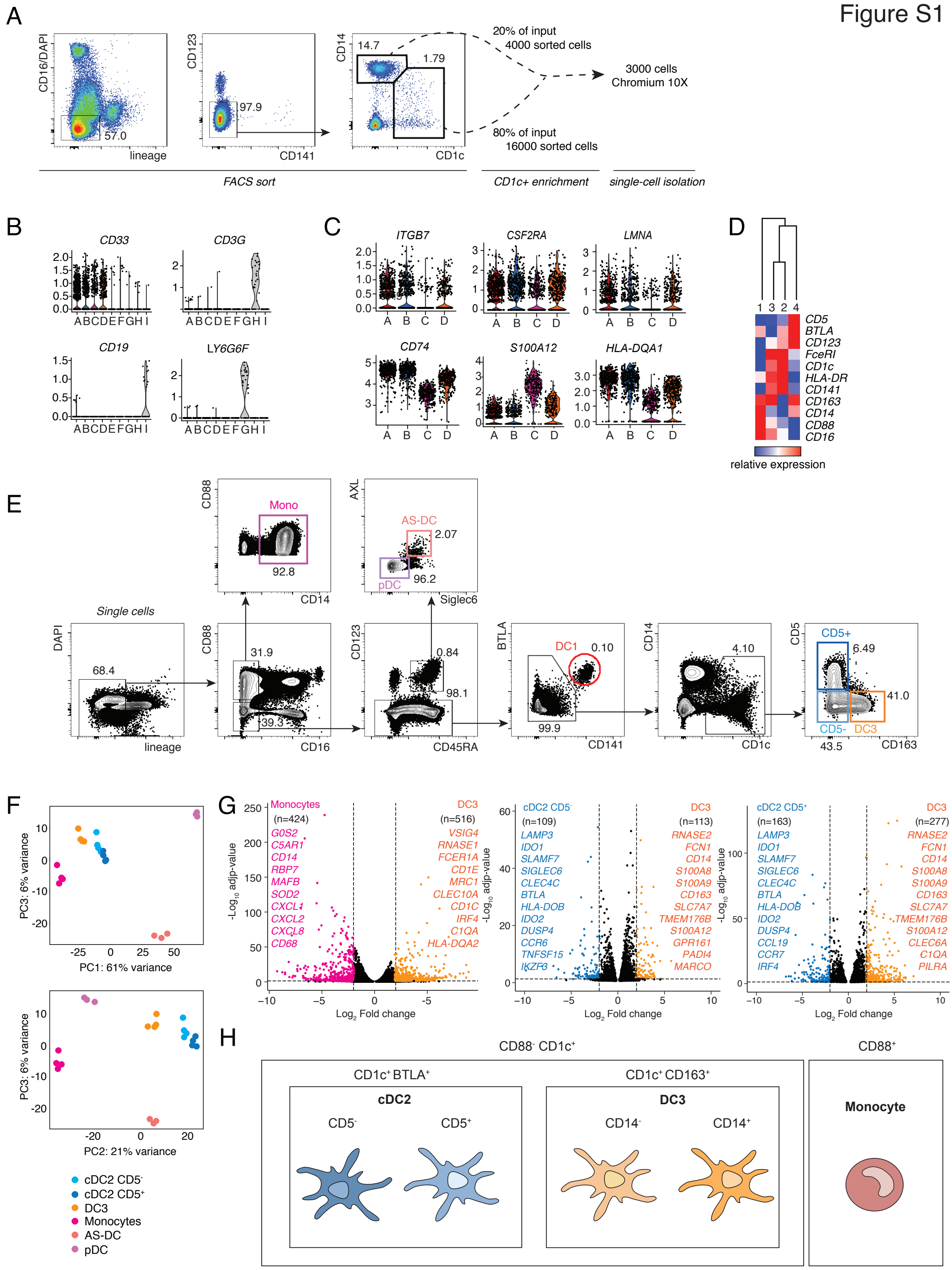

Figure S1. Related to Figure 1

(A) Gating strategy used to identify CD14$^{+/-}$ and/or CD1c$^{+/-}$ cells for scRNAseq analysis. In order to enrich for CD1c-expressing cells, CD14^{+} monocytes and CD1c^{+}CD14$^{+/-}$ cells from 3

healthy donors were sorted separately and then pooled together in a 20:80 ratio of monocytes versus $CD1c^+$ cells. 3000 cells from the obtained suspension were loaded into a Chromium 10X chip for droplet-based single-cell isolation. (B) Violin plots illustrating expression probability distributions across clusters of lineage-specific markers used to discriminate CD33-expressing myeloid clusters (A, B, C and D) from additional contaminating subsets (clusters E, F, G, H and I). (C) Violin plots illustrating expression probability distributions of representative DEG across clusters A, B, C and D (226 total DEG). (D) Heat map displaying relative expression of markers detected by flow cytometry on subsets 1, 2, 3 and 4 identified by FlowSOM analysis. (E) Gating strategy used to define the blood cell subsets sorted for the bulk RNAseq. (F) PCA of DEG for bulk sequenced mononuclear phagocyte populations. Displayed principal components 1 versus 3 and 2 versus 3. (G) Volcano plots showing the DEG between bulk sequenced DC3 as compared to monocytes or both $CD5^+$ and $CD5^-$ cDC2 cells. Genes with $Log_2(FC)>\pm2$ and FDR adjusted p value less than 0.05 were considered significant. Genes in bold are differentially expressed in $CD5^+$ and $CD5^-$ cDC2s when compared to DC3s. (H) Graphical summary of the blood $CD1c^+$ and monocyte cells.

Figure S2

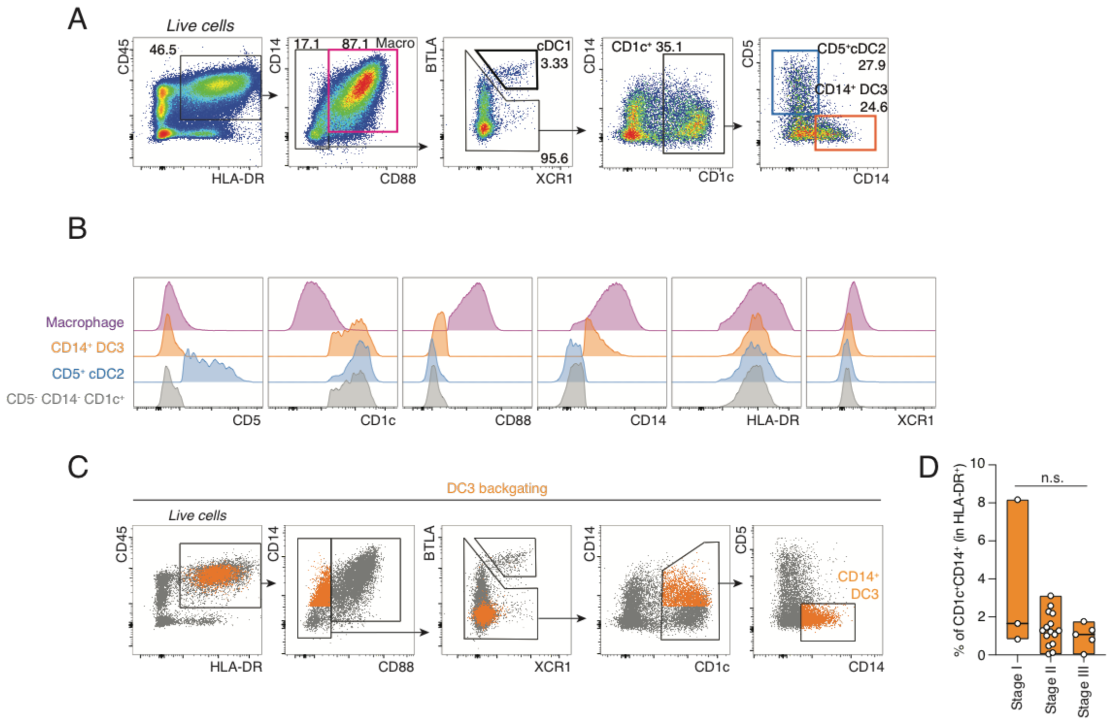

Figure S2. Related to Figure2

(A) Full gating strategy used to define macrophages, cDC1s, CD5$^+$ cDC2s and CD14$^+$ DC3s in human breast cancer primary tumors. (B) Histograms showing the expression of CD5, CD1c, CD88, CD14, HLA-DR and XCR1 in macrophages, cDC1s, CD5$^+$ cDC2s, CD14$^+$ DC3s and CD5$^-$CD14$^-$CD1c$^+$ in human breast cancer primary tumors. (C) Backgating of the CD14$^+$ DC3s in human breast cancer primary tumors. (D) Quantification of the CD1c$^+$CD14$^+$ cells in the different stages of human breast cancer primary tumor (stage I n=3; stage II n=15; stage III n=5).

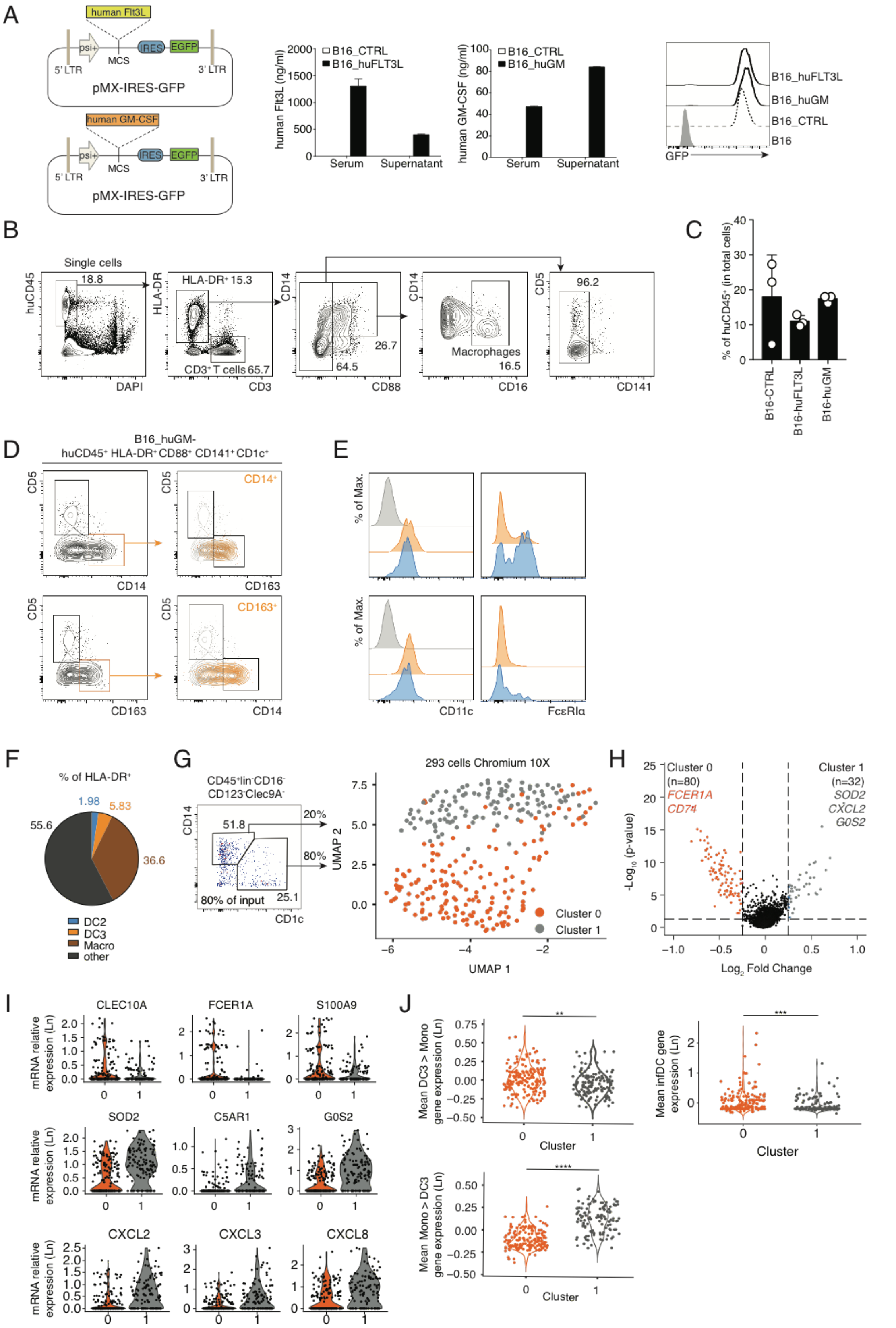

Figure S3. Related to Figure 3

(A) Schematic of plasmids used to generate B16 melanoma cell lines expressing human

FLT3L (B16_huFL) and human GM-CSF (B16_huGM). The production of human cytokines was validated by ELISA performed on the supernatant of cultured cells as well as on the serum of tumor-bearing mice. The expression of a reporter gene (GFP) was assessed by flow cytometry. (B) Gating strategy used to define macrophages, $CD3^+$ T cells and $huCD45^+HLA\text{-}DR^+CD88^-CD141^-$ cells in B16_huGM metastatic lung at day 9. (C) Bar graph summarizing the frequency of total human $CD45^+$ cells isolated from B16_CTRL, B16_huFLT3L or B16_huGM metastatic lung at day 9 (n=3). (D) Backgating of the $CD1c^+CD163^+CD5^-$ and $CD1c^+CD14^+CD5^-$ in the B16_huGM metastatic lung (related to fig. 3C). (E) Histograms showing the expression of CD11c and FcεRI on cDC2 (blue) and DC3 (orange) and $CD3^+$ T cells (grey) (related to fig. 3C). (F) Pie chart summarizing the average frequency of each subset within $HLA\text{-}DR^+$ cells. (G) Gating strategy used to define $CD14^{+/-}$ and/or $CD1c^{+/-}$ cells for scRNAseq analysis. Single cells were isolated using a droplet-based approached and sequenced. Dimensionality reduction of scRNA-seq data was performed using UMAP. Clusters 0 and 1 were identified using SNN clustering algorithm. Each dot represents an individual cell (n=293). (H) Volcano plots showing the DEG between clusters 0 and 1 identified in (F). Genes with $Log_2(FC)>\pm0.5$ and FDR adjusted p value less than 0.05 were considered significant. (I) Violin plots illustrating expression probability distributions across the two clusters of representative genes (out of 112 differentially expressed genes). (J) Expression distribution across clusters 0 and 1 of gene signatures identified in Villani et al., 2017. Signatures were defined as mean expression of discriminative markers for DC3 and $CD14^+$ monocytes within the $lin^-CD14^{+/-}$ cells (Villani et al., 2017).

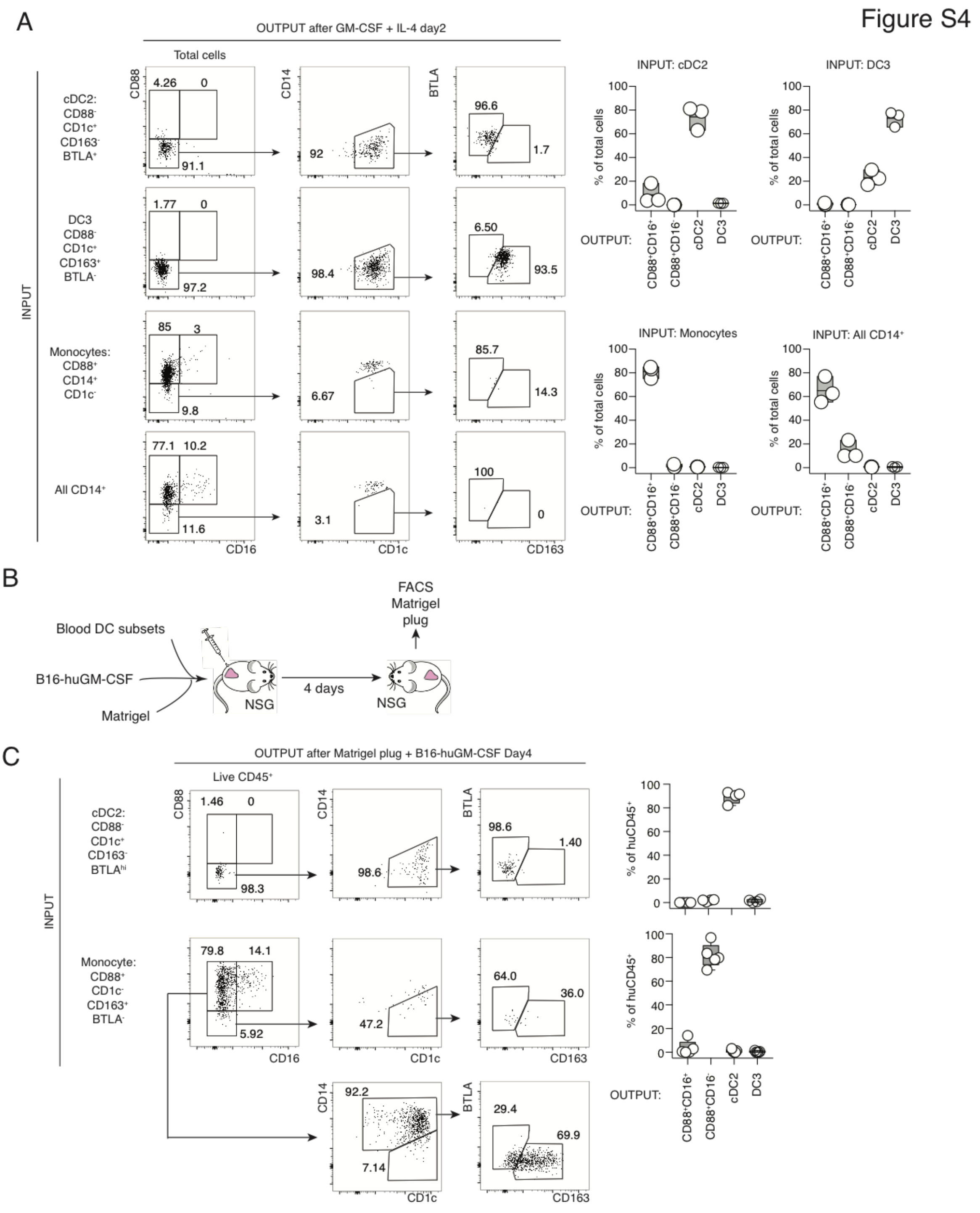

Figure S4, related to Fig.3

(A) Flow cytometry analysis of flow cytometry-sorted cDC2, DC3, CD88+CD14+ monocytes and all CD14+ cells after 2 days of culture with 100ng/ml recombinant GM-CSF and 50ng/ml recombinant IL-4. Bar graphs show the frequency of output cells within the total live cells (n=3 healthy donors). (B) Experimental strategy for in vivo plug. flow cytometry-sorted blood myeloid cells were injected subcutaneously along with B16-huGM in a basement membrane matrix (Matrigel) preparation. (C) Flow cytometry analysis of flow cytometry-sorted cDC2 and CD88+CD14+ monocytes after 4 days in B16-huGM containing Matrigel plug. Bar graphs show the frequency of output cells within the total huCD45+ cells (n=4-5 healthy donors).

Figure S5

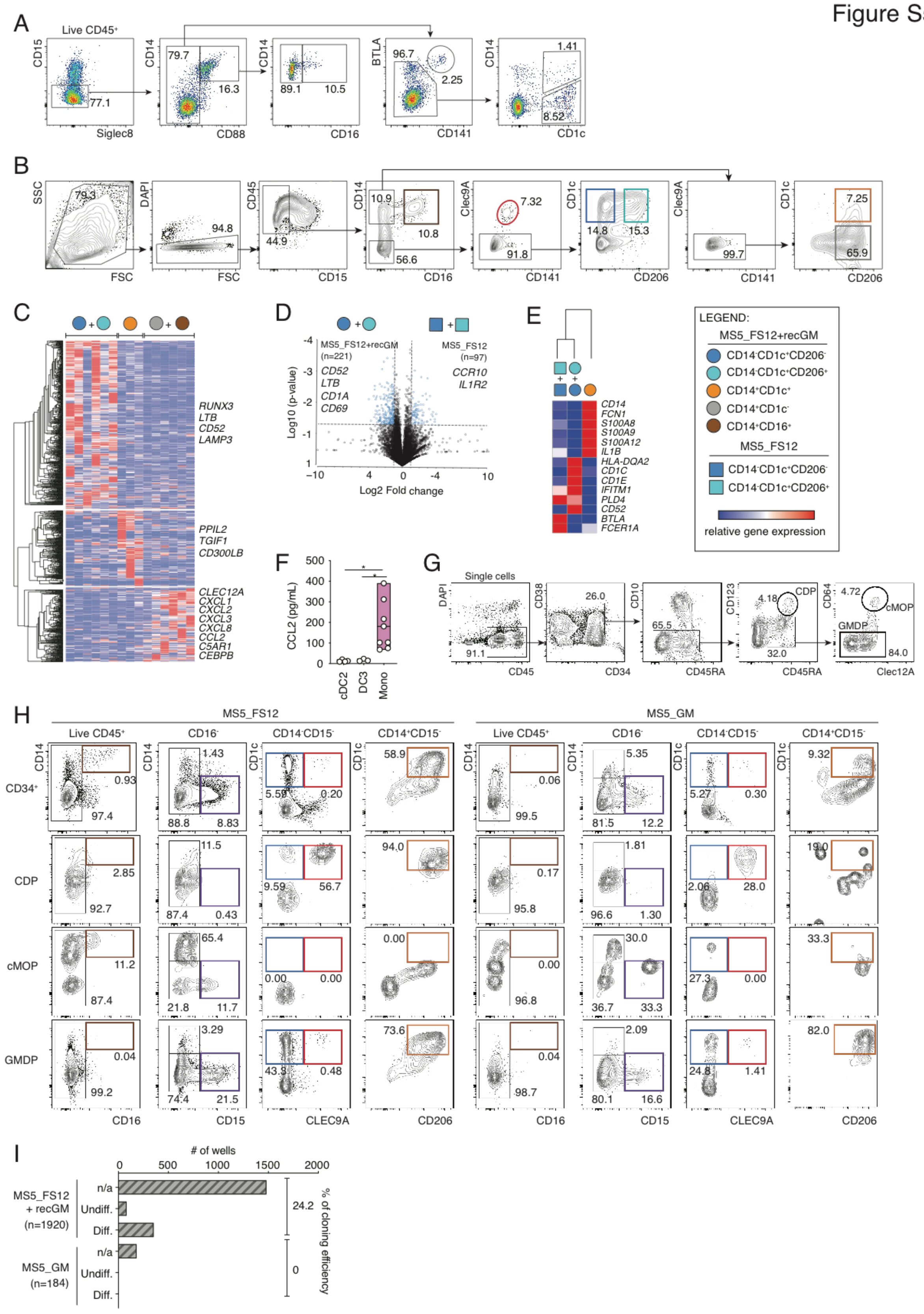

Figure S5. Related to Figures 4 and 5

(A) Full gating strategy CD1c^{+}CD14^{+} and the CD1c^{+}CD14^{-} after coculture of human cord blood-derived CD34^{+} with MS5_FS12. (B) Gating strategy used to flow cytometry-sort for bulk RNAseq analysis of *in vitro*-differentiated macrophages (brown), monocytes (grey),

cDC1s (red), CD206 +/- cDC2s (blue and turquoise) and DC3s (orange) after coculture of human cord blood-derived CD34^{+} with MS5_FS12 in presence of GM-CSF. (C) Heat map of discriminative gene sets based on Signal to noise ratio across the different cell types for CD14^{-}CD1c^{+}CD206$^{+/-}$ cDC2-like cells (blue and turquoise), CD14^{+}CD1c^{+} DC3-like cells (orange), CD14^{+}CD16$^{+/-}$CD1c^{-} macrophage-like cells (grey and brown). (D) Volcano plots showing the DEG between *in vitro* generated cDC2 cells cocultured with MS5_FS12+recGM (blue and turquoise circles) as compared to cDC2-like cells cocultured with MS5_FS12 (blue and turquoise squares). Genes with $Log_2(FC)>\pm2$ and FDR adjusted p value less than 0.05 were considered significant. (E) Heat map showing the relative expression of markers used for subset identification in Fig. 1. (F) Quantification of CCL2 secreted by cDC2-, DC3- and Macro-like *in vitro* generated cells in response to overnight stimulation with a cocktail of TLR agonists (n=3-8; line represent median; *$p<0.05$, one-way ANOVA test). (G) Gating strategy defining CDP, cMoP and GMDP within CD34^{+} cord blood derived HSPC. (H) Gating strategy to identify CD14^{+}CD16^{+} macrophages (brown), CD14^{-}CD15^{+} granulocytes (purple), CD14^{+}CD1c^{+} DC3 (orange), CD14^{-}CD1c^{+}Clec9A^{-} cDC2 (blue) and CD14^{-}CD1c^{+}Clec9A^{+} cDC1 (red) in 7 days cultures of CDP, cMoP and GMDP with MS5_FS12 and MS5_GM. (I) Bar graph summarizing the number of wells resulting in differentiated cells from single CD34^{+}CD38^{+}CD45RA^{+}CD123^{-}CD64^{-} cells cocultured MS5_FS12+recGM-CSF or MS5_GM and percentage of cloning efficiency. n/a (not available) corresponding to empty wells at the read out.

Figure S6

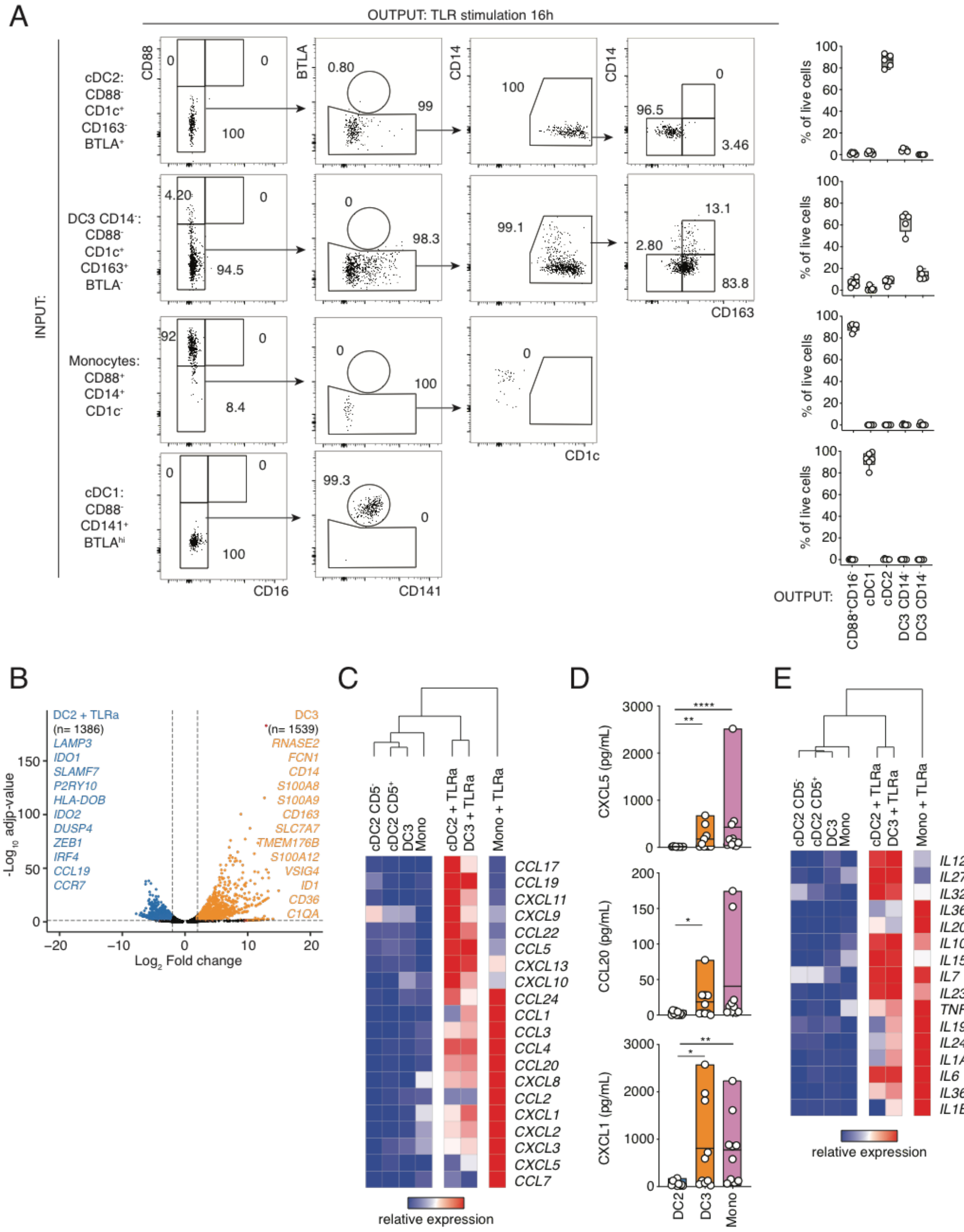

Figure S6 related to Fig. 6

(A) Flow cytometry analysis of flow cytometry-sorted cDC1, cDC2, DC3 and CD88^{+}CD14^{+} monocytes after 16 hours of activation with TLR agonists (25µg/ml PolyI:C, 1µg/ml R848 and 10ng/ml LPS). Bar graphs show the frequency of output cells within the total live cells (n=6 healthy donors). (B) Volcano plots showing the DEG between bulk sequenced TLR agonist stimulated cDC2 as compared to not stimulated DC3. Genes with Log$_2$(FC)>±2 and FDR adjusted p value less than 0.05 were considered significant. (C) Heat map showing the relative gene expression of chemokines in TLR agonist cocktail stimulated (25µg/ml PolyI:C, 1µg/ml R848 and 10ng/ml LPS) or not stimulated mononuclear phagocyte populations. (D) Quantification of chemokines secreted by cDC2, DC3 and monocytes in response to overnight stimulation with a cocktail of TLR agonists (n=9 healthy donors; line represent median; *p<0.05, **p<0,01, ****p<0.0001, one-way ANOVA test). (E) Heat map showing the relative gene expression of cytokines in TLR agonist cocktail stimulated or not stimulated mononuclear phagocyte populations.

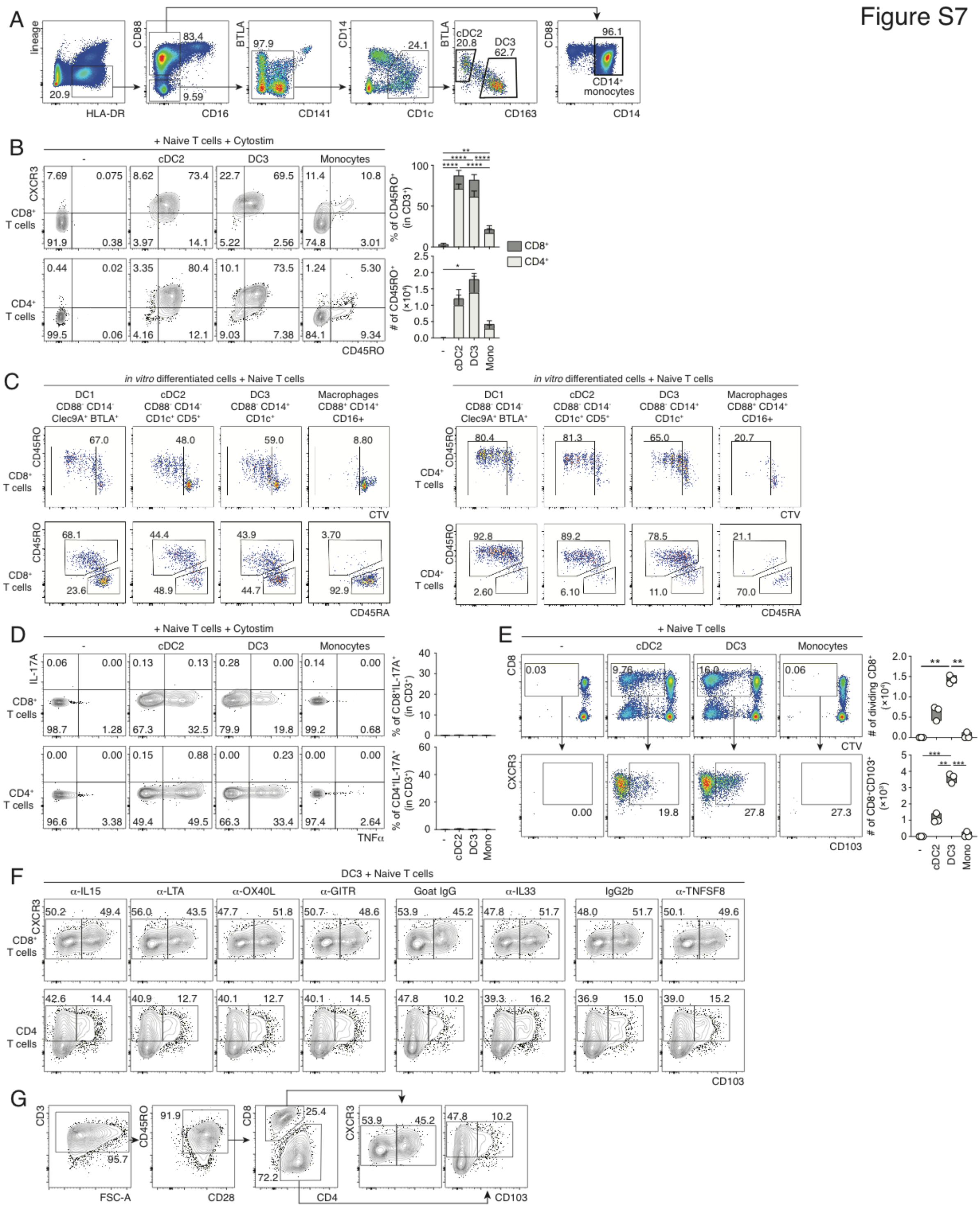

Figure S7, related to Fig. 7

A) Gating strategy used to flow cytometry-sort blood cDC2, DC3 and monocytes for T cell coculture. (B) and (D) Representative flow cytometry plots and quantification of CD4$^+$ and CD8$^+$ naïve T cells cultured for 5 days with flow cytometry-sorted blood cDC2, DC3 or CD14$^+$ monocytes after overnight activation with TLR agonists (25µg/ml PolyI:C, 1µg/ml R848 and 10ng/ml LPS) in the presence of a synthetic superantigen (Cytostim). Absolute numbers and frequencies of CD45RO$^+$ differentiated T cells (B), cytokines-producing activated T cells (D). (n=5 healthy donors in 5 independent experiments, line represent median; *p<0.05, **p<0.01, ***p<0.001 and ****p<0.0001; one-way ANOVA test). (C)

Flow cytometry plots of CTV-labeled CD4$^+$ and CD8$^+$ naïve T cells cultured for 5 day with flow cytometry-sorted DC1, cDC2, DC3 and macrophages generated *in vitro*, on MS5-FL for cDC1 and cDC2 or MS5-GM for DC3 and macrophages, after overnight activation with TLR agonists (25µg/ml PolyI:C, 1µg/ml R848 and 10ng/ml LPS) in the presence of a synthetic superantigen (Cytostim). CTV dilution and expression of CD45RA and CD45RO are shown (n=1 in 1 experiment). (E) Representative flow cytometry plots and quantification of CTV-labelled CD8$^+$ naïve T cells cultured for 5 days with flow cytometry-sorted blood cDC2, DC3 or CD14$^+$ monocytes after overnight activation with TLR agonists (25µg/ml PolyI:C, 1µg/ml R848 and 10ng/ml LPS). Proliferation (CTV dilution) and CD103 expression on CD8$^+$ T are shown. (n=3 healthy donors in 3 independent experiments, line represent median; **$p<0.01$ and ***$p<0.001$; one-way ANOVA test). (F) Flow cytometry plots showing CD103 expression on CD8$^+$ naïve T cells cocultured with flow cytometry-sorted blood DC3 after overnight activation with TLR agonists (25µg/ml PolyI:C, 1µg/ml R848 and 10ng/ml LPS) in the presence of 10µg/ml of neutralizing antibodies or their respective isotype controls (n=1 healthy donor in 1 experiment). (G) Gating strategy used to flow cytometry-sort CD103$^-$ and CD103$^+$CD8$^+$ T cells cultured for 5 days with flow cytometry-sorted blood DC3 after overnight activation with TLR agonists (25µg/ml PolyI:C, 1µg/ml R848 and 10ng/ml LPS).

	Code patient	Age	HISTO.	GRADE	SIZE	pTNM	ER (%)	PR (%)	HER2	Ki67 (%)
Primary tumor	1	57	Ductal	3	32 mm	pT2 N0	100	15	neg	60
Primary tumor	2	71	Ductal	2	18 mm	pT1c N0	90	10	neg	15
Primary tumor	3	72	Lobular	2	5 mm	pT2 N1a	80	80	neg	20
Primary tumor	4	41	Mixte	2	4 nodules (5 to 30 mm) and multiples satellite nodules (1 to 4 m	pT2m N1	70	60	N/A	80
Primary tumor	5	55	Ductal	2	3 to 16 mm	pT1cm N1	95	1	neg	15
Primary tumor	6	76	Ductal	3	22 mm	pT2 N1	100	0	neg	35
Primary tumor	7	61	Ductal	3	32 x 25 mm	pT2 N0	100	60	neg	40
Primary tumor	8	70	Ductal	3	30 + 26 + 18 mm	pT2m N1	100	80	N/A	30
Primary tumor	9	55	Lobular	3	25 mm	pT2m N0	70	5	neg	35
Primary tumor	10	59	Ductal	2	15 mm	pT1c N1	0	0	pos	25
Primary tumor	11	48	Lobular	2	60 mm and multiples satellite nodules (1 to 5 mm)	pT3 N1	100	50	neg	35
Primary tumor	12	58	Ductal	3	26 x 22 mm + one satellite nodule (2 mm)	pT2 N0	95	85	neg	25
Primary tumor	13	84	Ductal	1	22 mm	pT2 N2	100	80	neg	18
Primary tumor	14	82	Ductal	2	12 + 16 mm	pT1c N1	100	10	neg	5
Primary tumor	15	37	Multifocal	2	35 + 22 + 15 + 6 mm	pT2 N1	100	5	neg	25
Primary tumor	16	70	Ductal	2	21 mm	pT2m N0	100	100	neg	15
Primary tumor	17	77	Carcinoma	2	65 mm	pT3 N2	100	80	neg	2
Primary tumor	18	46	Ductal	3	40 mm	pT2 N1	50	80	neg	60
Primary tumor	19	54	Mixte	3	25 mm	pT2 N0	100	N/A	neg	20-25
Primary tumor	20	63	Ductal	2	40 mm	pT2 N1a	100%	10%	neg	50
Primary tumor	21	55	Lobular	2	32 mm	pT2 N0	100	10	neg	40
Primary tumor	22	81	Lobular	2	60 mm	pT3 N3a	100	80	neg	17
Primary tumor	23	48	Lobular	2	6 mm	pT3 N1a	70	40	N/A	10
Primary tumor	24	40	Ductal	2	13 mm	pT1c N0	100	90	neg	5
Primary tumor	25	51	Ductal	3	53 mm	pT3 N0	100	0	neg	30
Primary tumor	26	54	Ductal	2	16 mm	pT1c N0	80	10	neg	5
Primary tumor	27	62	Ductal	2	17 mm	pT1c N1	95	85	neg	22
Primary tumor	28	63	Ductal	2	40 mm	pT2 N1a	100	10	neg	50
Invaded lymph node	1	52	Ductal	2	17 mm	pT1c	100	100	neg	11
Invaded lymph node	2	76	N/A	3	32 mm	pT2N2	100	10	neg	30
Invaded lymph node	3	61	Ductal	3	25 mm	pT2N1a	5	0	pos	60

Table S2: Clinical data for the patients with primary breast tumors and invaded lymph nodes, Related to Figures 2 and 7.
